# *Devario* in Bangladesh: Species diversity, sibling species, and introgression within danionin cyprinids (Teleostei: Cyprinidae: Danioninae)

**DOI:** 10.1371/journal.pone.0186895

**Published:** 2017-11-22

**Authors:** Sven O. Kullander, Md. Mizanur Rahman, Michael Norén, Abdur Rob Mollah

**Affiliations:** 1 Department of Zoology, Swedish Museum of Natural History, Stockholm, Sweden; 2 Department of Zoology, University of Dhaka, Dhaka, Bangladesh; SOUTHWEST UNIVERSITY, CHINA

## Abstract

Four species of *Devario* are recorded from Bangladesh: *D*. *aequipinnatus*, *D*. *anomalus*, *D*. *coxi*, new species, and *D*. *devario*. *Devario aequipinnatus* has a wide distribution in northern India and Bangladesh. *Devario coxi*, from southeastern Bangladesh near Cox’s Bazar, differs from *D*. *aequipinnatus* in mtDNA (*COI*, *p-*distance 1.8%), colouration, proportional measurements, and meristics. The minor morphological differences and low frequency of overlapping meristics suggest relatively recent separation of *D*. *coxi* from other *D*. *aequipinnatus*. *Devario anomalus* occurs only in southeastern Bangladesh and is here reported from localities in addition to the type locality. It differs from the similar *D*. *xyrops* in adjacent Myanmar by slender body shape and by 2.3% *p-*distance in the *COI* gene. Specimens of *D*. *anomalus* from the Sangu River were found to have the mitochondrial genome of *D*. *aequipinnatus* from Bangladesh, but agree with other *D*. *anomalus* in the nuclear *RAG1* gene. *Devario devario* has a wide distribution on the Indian Peninsula and border regions; in Bangladesh it is restricted in distribution to the Ganga, Brahmaputra, and Meghna drainages. Reports of *D*. *assamensis* and *D*. *malabaricus* from Bangladesh are misidentifications. *Perilampus ostreographus* M’Clelland, 1839, is tentatively synonymized with *D*. *aequipinnatus*. Phylogenetic analysis of 14 species of striped devarios based on the *COI* gene results in a polytomy with four unresolved clades. *Devario deruptotalea* from the Chindwin basin is the sister group of *D*. *aequipinnatus*+*D*. *coxi*. *Devario devario* is the sistergroup of *D*. *xyrops*+*D*. *anomalus*.

## Introduction

The cyprinid genus *Devario* Heckel, 1843, includes about 38 valid species with a combined distribution covering freshwater habitats in most of tropical South and Southeast Asia from the Indus River drainage eastward to the Mekong River drainage [1–3]. They are relatively small species from about 50 to 100 mm body length, and distinguished from other danionine cyprinids by the combination of rounded abdominal midline (vs. keeled), neuromast-filled frontal grooves, minute or absent maxillary barbel, a dark spot close to the gill cleft, and in most species a short sharp projection from the first infraorbital [4–5]. Species of *Devario* are mainly found in small schools in streams at higher elevations. The genus was partially revised by Fang [1,4] and Fang et al. [5]. Kottelat [6] suggested splitting the genus in two, recognizing *Devario* for species most of which share a predominantly horizontally striped colour pattern, and putting the rest in *Inlecypris* Howes, 1980, characterized by dark vertical bars, but here we consider *Devario* in the wider sense of Fang et al. [5]. The majority of the nominal species have not been subject to recent revision.

Five species of *Devario* have been reported from Bangladesh, including *D*. *aequipinnatus* (M’Clelland, 1839), *D*. *anomalus* Conway, Mayden & Tang, 2009, *D*. *assamensis* (Barman, 1984), *D*. *devario*, and *D*. *malabaricus* (Jerdon, 1849) [7–11]. *Devario ostreographus* (M’Clelland, 1839), described from “Assam, and some parts of Bengal”, has not been recorded specifically from Bangladesh, but it was treated as removed from the synonymy of *D*. *devario* by Conway et al.[9] and discussed by them in the context of Bangladeshi *Devario*. Actually, species described by Hamilton [12] and M’Clelland [13] as coming from Bengal or Assam may have been obtained or observed in parts of British India that are within present-day Bangladesh as well as within the present Indian states of West Bengal and Assam.

*Devario devario* is undisputed as a Bangladeshi species. It was described in 1822 by Francis Hamilton [12] from “rivers and ponds of Bengal”, an area that includes most of present-day Bangladesh. In a manuscript produced between 1807 and 1813 and reproduced by Day [10], Hamilton recorded *D*. *devario* with the local names Chhepká in Rangpur District (present Rangpur Division in Bangladesh), Bánspátá in Lakshmípur District (now Laksmipur District in southern Bangladesh), and Deborí in Dinájpur District (now split between India and Bangladesh) [10]. He also recorded *D*. *devario* with the local name Jharaingi from the Gorakhpur District in India [9]. Hamilton [12] reported *D*. *devario* with the local name Debari, as a “pretty common fish in the rivers and ponds of Bengal.” Remarkably, Hamilton did not report observation of any other species of *Devario*. Except for *D*. *devario*, none of the species descriptions in [12], or Hamilton drawings published later [13–16] can be identified as a species of *Devario*.

There are numerous published records of *Devario aequipinnatus* from India and neighbouring countries (e.g.,[17]), but the taxonomic status of *D*. *aequipinnatus* has never been satisfactorily resolved, and the name has been used alternating with *D*. *malabaricus* as a convenient label for striped species of *Devario*. *Devario malabaricus* is also a species in need of revision, and it seems unlikely that it would have a distribution extending far away from the type locality on the southwestern coast of India. *Devario assamensis* is a deep-bodied species otherwise only known from three specimens from the Brahmaputra River in Assam, India [17–19]. *Devario anomalus* was described based on specimens collected near Cox’s Bazar in southeastern Bangladesh [9], and is doubtlessly a valid, locally endemic species. It is, however, very similar to *D*. *xyrops* Fang & Kullander, 2009, from the adjacent western slope of the Rakhine Yoma in Myanmar [20]. Apparently, with the exception of *D*. *devario*, the reported Bangladeshi assemblage of *Devario* offers several problem taxa, some of them highly significant for the taxonomy of *Devario*.

Recent collections from Bangladesh confirm three of the species reported earlier, and adds an undescribed species. Here we provide distribution data, morphological diagnoses, DNA barcodes, and images of all species of *Devario* recorded from Bangladesh, and review the status of all names applied on Bangladeshi *Devario*.

## Material and methods

Specimens are kept in the following collections: Zoology Department, University of Dhaka, Dhaka (DU); Swedish Museum of Natural History, Stockholm (NRM); University of Michigan Museum of Zoology, Ann Arbor (UMMZ). Some comparative material is kept in the Natural History Museum, London (BMNH), Kansas University Biodiversity Institute and Natural History Museum, Lawrence (KU) Lawrence, Naturmuseum Senckenberg, Frankfurt/Main (SMF) Florida Museum of Natural History, Gainesville, (UF) or the Zoological Survey of India, Kolkata (ZSI). Measurements were taken with digital calipers to a precision of 0.1 mm. Counts and measurements were made according to Fang [21] and Kullander [22]. Colour pattern terminology follows Fang [23], with modifications for special markings as explained by Kullander [22]. Horizontal stripes and interstripes are identified by alphanumeric annotations. Fin-ray and vertebral counts were recorded from X-radiographs made with a Philips MG-105 low voltage X-ray unit and Kodak X-Omat V plates, or as digital images with a Kevex 130kVP microfocus X-ray source with a Samsung/Rayence 17x17 inch DR panel. Statistics were calculated using SYSTAT v. 13 [24]. The paired supraorbital (actually frontal) groove identified by Fang [4] as a characteristic of *Devario* and other genera of Devarionini and packed with neuromasts, is here referred to as neuromast grooves; an additional pair of short curved grooves is located anterior to the nostril and are here referred to as rostral neuromast grooves. Juvenile specimens preserved whole for DNA analyses were not measured.

655 basepairs from the 5’ end of the mitochondrial cytochrome-oxidase subunit 1 (*COI*, or *COX1*) gene was sequenced from 38 morphologically identified specimens of *Devario* plus two outgroup species (see Table 1 for voucher information and GenBank accession numbers). This fragment was chosen because it is the standard DNA barcoding fragment recommended by the Barcode of Life consortium [25]. To investigate possible hybridization a 1565 bp fragment of the nuclear recombination activating protein 1 (*RAG1*) gene, exon 3, was sequenced from a subset of 8 selected individuals of *Devario* plus three outgroup species.

**Table 1:** New sequences generated.

Species	Voucher Catalog Number (tissue number)	Locality	*COI* GenBank Accession no.	*RAG1* GenBank Accession no.
*Danio dangila*	NRM 49818 (1217)	Aquarium	–	MF991141
*Danio rerio*	NRM 41663 (63)	India: Assam: Dibrugarh	–	MF991142
*Devario aequipinnatus*	NRM 42646 (1434)	India: Assam: Dibru River	MF172749	MF172737
*D*. *aequipinnatus*	47425 (7184)	India: Meghalaya: Jatinga	MF172748	MF172736
*D*. *aequipinnatus*	NRM 51438 (7186)	India: Meghalaya: Makrei	MF172747	–
*D*. *aequipinnatus*	NRM 66258 (9865)	Bangladesh: Madobkundo Falls	MF172750	–
*D*. *aequipinnatus*	NRM 66259 (9866)	Bangladesh: Madobkundo Falls	MF172750	–
*D*. *aequipinnatus*	NRM 66705 (10126)	Bangladesh: Madobkundo Falls	MF172745	–
*D*. *aequipinnatus*	DU6253	Bangladesh: Momahaya Hill	MF172751	–
*D*. *aequipinnatus*	NRM 66832 (10198)	Bangladesh: Kaptai	MF172744	–
*D*. *aequipinnatus*	NRM 66813 (10223)	Bangladesh: Chittagong	MF172754	–
*D*. *aequipinnatus*	DU 6188	Bangladesh: Khagrachori	MF172753	–
*D*. *aequipinnatus*	DU 6244	Bangladesh: Khagrachori	MF172752	–
*D*. *anomalus*	NRM 67936 (10786)	Bangladesh: Mrong Bazar	MF172758	MF172740
*D*. *anomalus*	NRM 67937 (10787)	Bangladesh: Mrong Bazar	MF172757	MF172739
*D*. *anomalus*	NRM 65562 (9874)	Bangladesh: Cox’s Bazar	MF172756	MF172738
*D*. *anomalus*	NRM 67399 (10659)	Bangladesh: Cox’s Bazar	MF172755	–
*Devario browni*	NRM 58623 (6225)	Myanmar: near Taunggyi	MF172759	–
*Devario browni*	NRM 58624 (6226)	Myanmar: near Taunggyi	MF172760	–
*D*. *chrysotaeniatus*	32302 (54)	China: Yunnan: near Mengla	MF172761	–
*D*. *chrysotaeniatus*	NRM 32302 (131)	China: Yunnan: near Mengla	MF172762	–
*D*. *coxi*	DU 9003 (10543)	Bangladesh: Cox’s Bazar	MF172780	MF172742
*D*. *coxi*	NRM 67379 (10544)	Bangladesh: Cox’s Bazar	MF172779	–
*D*. *deruptotalea*	NRM 51359 (7069)	Myanmar: near Tamu	MF172763	–
*D*. *devario*	NRM 66262 (9867)	Bangladesh: Dhaka	MF172764	–
*D*. *devario*	NRM 66260 (9870)	Bangladesh: Dhaka	MF172766	–
*D*. *devario*	NRM 66730 (10119)	Bangladesh: Tista barrage	MF172765	–
*D*. *fangae*	NRM 41677 (62)	Myanmar: Putao	MF172767	–
*D*. *gibber*	NRM 47158 (1301)	Laos: Xe Katam	MF172768	–
*D*. *gibber*	NRM 47158 (1303)	Laos: Xe Katam	MF172769	–
*D*. *kakhienensis*	NRM 32303 (132)	China: Da Ying Jiang River	MF172770	–
*D*. *laoensis*	NRM 52044 (4251)	Laos: Ban Sop Pong	MF172771	–
*D*. sp. cf. *myitkyinae*	NRM 59287 (6328)	Myanmar: Kalaymyo	MF172775	MF172741
*D*. sp. cf. *myitkyinae*	NRM 42628 (9765)	Myanmar: near Htamanthi	MF172778	–
*D*. sp. cf. *mysoricus*	NRM 57798 (5866)	India: Karnataka: Western Ghats	MF172778	–
*D*. sp. cf. *mysoricus*	NRM 57798 (5867)	India: Karnataka: Western Ghats	MF172777	–
*D*. sp. cf. *mysoricus*	NRM 57798 (5868)	India: Karnataka: Western Ghats	MF172776	–
*D*. *regina*	NRM 11885 (8870)	Thailand: Ka Chong	MF172772	–
*D*. *regina*	NRM 11879 (8864)	Thailand: Ranong	MF172773	–
*D*. *xyrops*	NRM 41674 (58)	Myanmar: near Taunggok	MF172781	MF172743
*Leptobarbus* sp.	NRM 51750 (1591)	Aquarium	MF991140	MF991143
*Malayochela maassi*	NRM 50167 (1341)	Aquarium	MF991139	–

DNA was extracted using a Thermo Scientific™ KingFisher™ Duo (Thermo Fisher Scientific) fully automated liquid-handling instrument, with the Thermo Scientific KingFisher Cell and Tissue DNA Kit (Thermo Fisher Scientific) and recommended protocol. PCR were performed with the puReTaq Ready-To-Go PCR kit (Amersham Biosciences AB, Uppsala, Sweden). The *COI* fragment was amplified using the fish barcoding primers Fish-F1 and Fish-R [26], with the PCR cycling: 94°C 4 min; 35 * (94°C 30s; 52°C 30s; 72°C 30s); 72°C 8 min). The RAG1 fragment was amplified and sequenced using the primers R1_Dan1f (TGGCCATAAGGGTMAACAC) and R1_4078r (TGAGCCTCCATGAACTTCTGAAGRTAYTT) (this study), with the following PCR cycling protocol: 5 min 94°C; 5 * (52°C 30s, 72°C 45s’); 30 * (50°C 30s, 72°C 45s); 72°C 7 min. PCR products were checked on agarose gel, and purified by adding 5uL of a mix consisting of 20% Exonuclease I (EXO) and 80% FastAP Thermosensitive Alkaline Phosphatase (Fermentas/Thermo Fischer Scientific, Gothenburg, Sweden) to each 25 μl PCR reaction, incubated at 37°C for 30 minutes, then heated to 80°C for 15 minutes.

Sequencing of both strands of all fragments was carried out by Macrogen Europe (Amstelveen, The Netherlands). All sequences were assembled and proofread in the software Geneious version 10 [27]. Geneious was used to calculate genetic distances (uncorrected pairwise *p*-distance, as recommended by [28], and the Geneious plug-in Species Delimitation [29] was used to calculate the probability of reciprocal monophyly under a model of random coalescence. MrBayes version 3.2 [30–31] was used to construct a phylogenetic hypothesis (2 million generations, GTR + Γ + I model), data partitioned by codon position; samples were taken every 1000 generations, and the first 25% of samples were discarded as ‘burn-in’. Convergence was checked with Tracer version 1.6 [32]. *Leptobarbus* sp. (Cyprinidae) was designated outgroup in all phylogenetic analyses.

### Ethics statement and collecting permit

Specimens used were available in museums, purchased from fishermen or markets; or collected in the wild using beach seine or hand net and euthanized through immersion in buffered tricaine-methanesulphonate (MS 222) until cessation of opercular movements plus an additional 30 minutes, in accordance with permits from the Swedish Environmental Protection Agency (dnr 412-7233-08 Nv) and the Stockholm Ethical Committee of the Swedish Board of Agriculture (dnr N 85/15). Collecting in Bangladesh was conducted under a permit to the University of Dhaka.

### Nomenclatural acts

The electronic edition of this article conforms to the requirements of the amended International Code of Zoological Nomenclature, and hence the nomenclatural acts contained herein are available under that Code from the electronic edition of this article. This published work and the new name and other nomenclatural acts it contains have been registered in ZooBank, the online registration system for the ICZN. The ZooBank LSIDs (Life Science Identifiers) can be resolved and the associated information viewed through any standard web browser by appending the LSID to the prefix “http://zoobank.org/”. The LSID for this publication is urn:lsid:zoobank.org:pub:DE58B775-E497-40B1-8466-7804DE5B3D89. The electronic edition of this work was published in a journal with an ISSN, and has been archived and is available from the following digital repositories: PubMed Central, LOCKSS.

### Comparative material

Specimens used by Kullander [2] and Fang & Kullander[20]. *Devario aequipinnatus*. India: BMNH 1860.3.19.815–817, 3, 48.0–65.6 mm SL.—BMNH 1889.2.1:1289–1291, 3, 50.2–68.7 mm SL.—CAS 128713, 1, 75.0 mm SL. West Bengal, Siliguri. Shaw & Shebbeare.—CAS 141133, 8, 46.8–72.3 mm SL, Kalimpong Duars & Siliguri Terai; S. L. Hora, Nov 1938.—CAS 141134, 2, 44.2–52.7 mm SL, Tista Bridge, Peshoke Jhara, 4 Feb 1931.—FMNH 2346. 1, 50.9 mm SL, Darjeeling. F. Day.—FMNH 51285, 1, 68.0 mm SL, Darjeeling: Sevoke. H. Stevens, 3 Nov 1930. NRM 42646 (T1434) 1, 46.4 mm SL. Brahmaputra River drainage, River Dibru 1 km upstream of Digholtarang; K.K. Lahkar, 25 Nov 1998,—NRM 47424, 1, 57.5 mm SL; NRM 47425 (T7184), 1, tissue; Meghalaya: Barak River drainage: Jatinganala River, Jatinga Village, south of Haflong, Cachar, H. Bleher, 24 Feb 2009.—NRM 51438 (T7186), 1, tissue, NRM 51439, 4, 39.3–47.3 mm SL, Meghalaya, Meghna River drainage, Tahangpraich River, near Makrei; H. Bleher, 22 Feb 2009.—NRM 52655, 3, 27.1–38.4 mm SL, Assam, Brahmaputra River drainage, Kalmoni River drainage, Rani Garden, Ranigodam, ornamental fish farm and tea estate (26°3′42″N, 91°37′54″), 26 Oct 2005, O. Åhlander et al.—NRM 52692, 3, 23.4–39.1 mm SL, Assam, Brahmaputra River drainage, Golaghat District, Hatikhuli Tea Estate, 26°34′42″N, 93°24′24″E), 24 Oct 2005, O. Åhlander *et al*.—Nepal:—KU 29524, 20, 34.8–42.8 mm SL, Sankhuwasabha, Tumlingtar just E of Tumlingtar (27.3199°N, 87.2083°E, 18 Aug 1996, D.R. Edds.—KU 29529, 3, 51.2–56.5 mm SL, Sankhuwasabha, Tumlingtar, at Sabha River confluence, 1 h walk S of Tumlingar (27.2717°N, 87.2083°E, D.R. Edds, 19 Aug 1996, D.R. Edds.—*Devario assamensis* (photographs and notes only): ZSI FF1861. 1, holotype of *Danio assamensis*, 62.2 mm SL. India: Assam: streamlets roundabout Tangla, Darrang district; 14 Nov 1939; S.L. Hora.—ZSI FF1862. 2, paratypes of *Danio assamensis*, 60.1–74.5 mm SL. Same data as ZSI FF1861.—*Devario devario*: NRM 26409, 2, 60.3–47.7 mm SL; India: West Bengal: Brahmaputra River drainage: Siliguri; R. Malaise, 15 Dec 1934.—SMF 8835, 4, 62.0–67.5 m SL; Assam or Bengal; J. McClelland, received 1844.—UMMZ 243656, 8, 47.2–56.1 mm SL, India, West Bengal, Ganga River drainage, Panga River at Krishibagan; Ng H.H. et al. 8 Apr 2004.—*Devario* sp.: CMK 7250, 2 47.2–54.3 mm SL, India, Maharashtra, Satara District, Ondishi Village, 3 km W at Dhom Reservoir, K.C. Jayaram, 2 May 1988.

## Results

### *Devario devario* (Hamilton, 1822)

Figs 1–3

**Fig 1:**
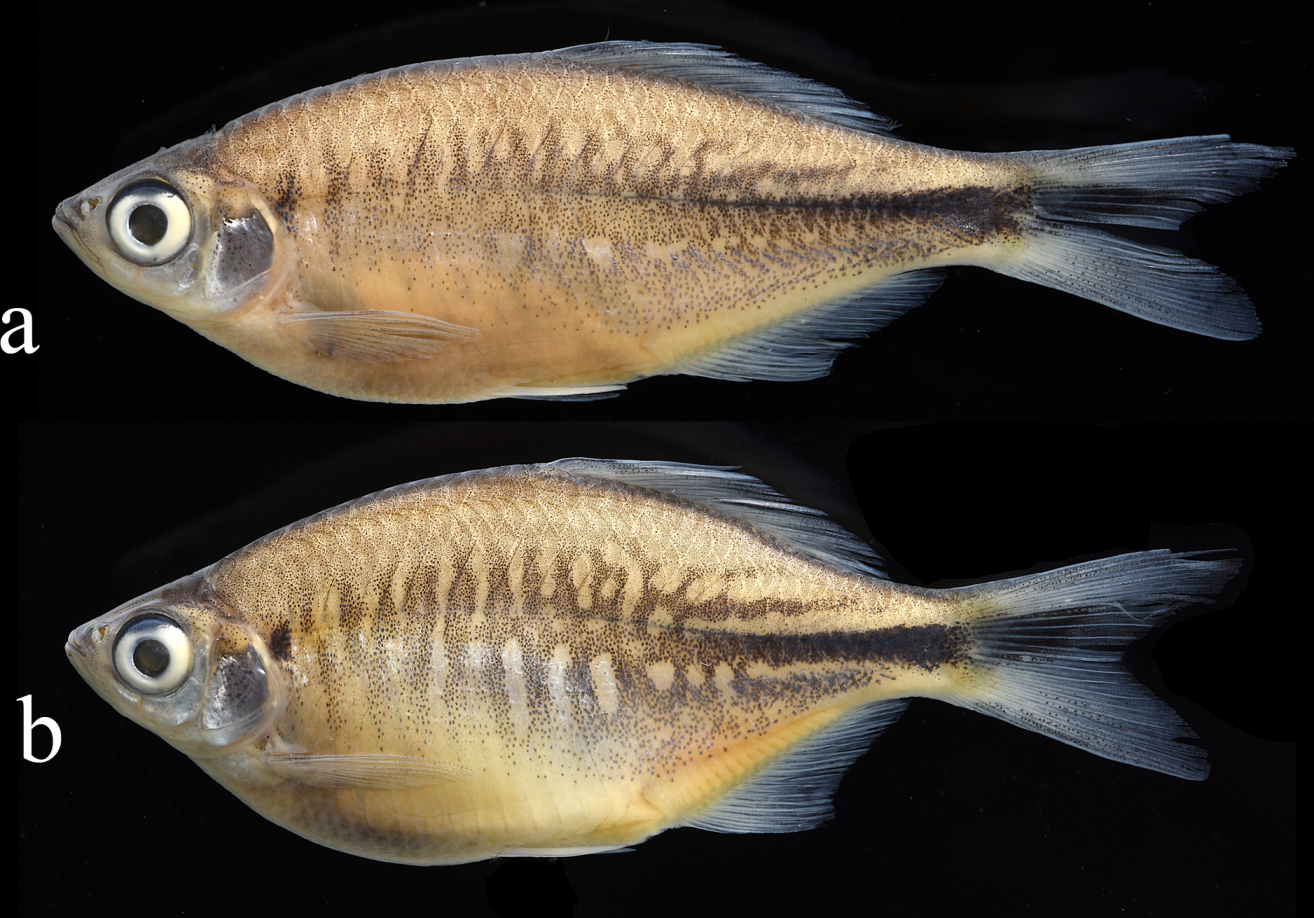
*Devario devario* from Bangladesh, Sylhet: Fenchuganj: a) adult male, NRM 68858, 47.4 mm SL; b) adult female, NRM 68858, 50.7 mm SL.

**Fig 2:**
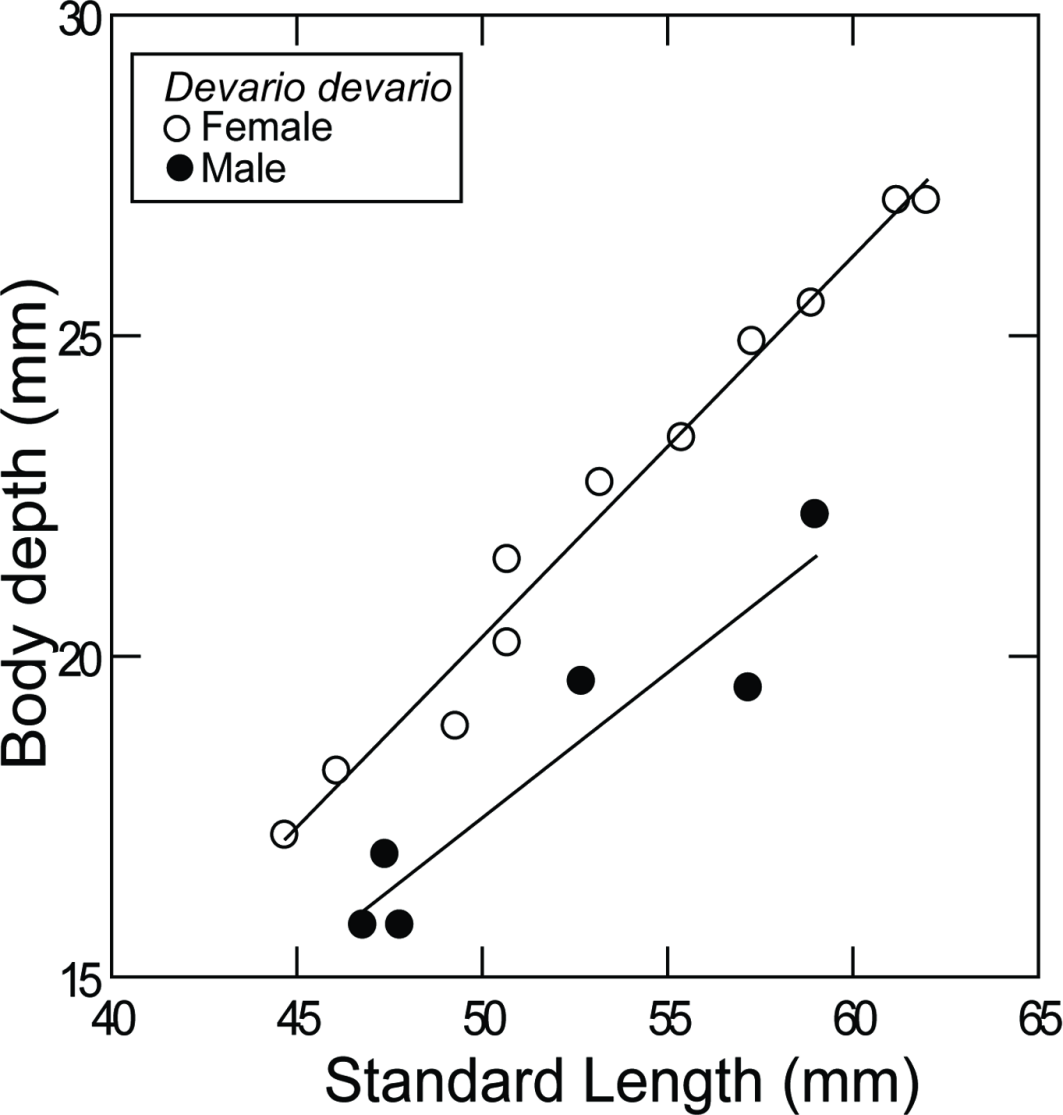
Sexual dimorphism in body depth in *Devario devario* from Bangladesh.

**Fig 3:**
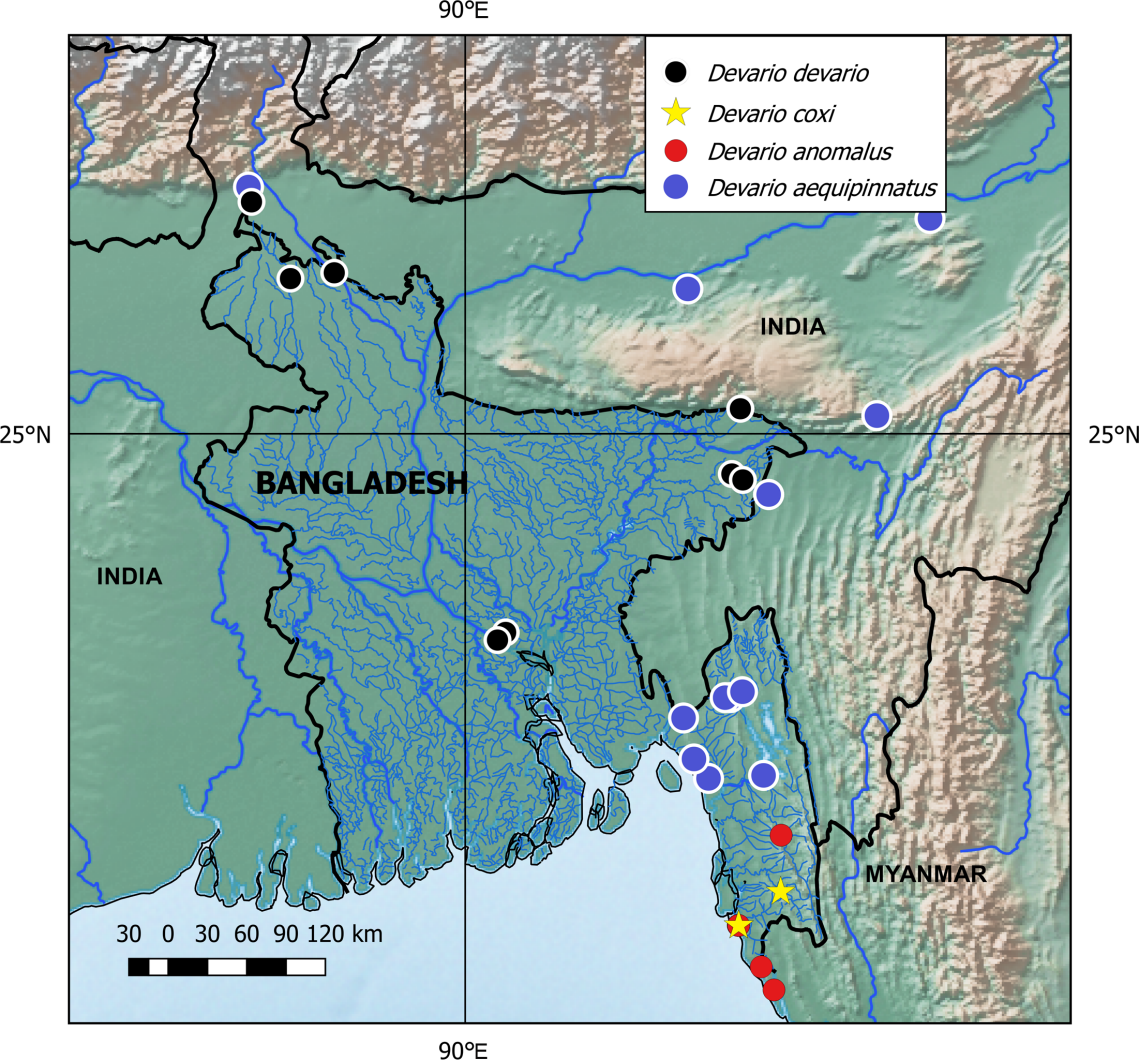
Collecting localities of species of *Devario* in Bangladesh and adjacent India.

*Cyprinus Devario* Hamilton, 1822[12], 341: 393 [in subgenus *Cabdio*] (rivers and ponds of Bengal) No types known.

*Devario Buchanani* Bleeker, 1860 [33]:283 (name only; unnecessary replacement name for *Cyprinus devario* Hamilton, 1822)

*Devario MacClellandi* Bleeker, 1860[33]:283 (name only; reference to pl. 56 [LVI in original], Fig 8 in M’Clelland, 1839[13], which corresponds to legend for pl. XLV, Fig 2*ß* on p. 314; unnecessary replacement name for *Cyprinus devario* Hamilton, 1822).

#### Material examined

All from Bangladesh. Dhaka Division: DU 6080 (EtOH), 2, 50.2–55.8 mm SL; DU 6082 (EtOH), 1, 48.9 mm SL; Dhaka fish market, Matikata Bazar; M.M. Rahman, 25 Jan 2015.—NRM 66260–NRM 66262, 3, tissue; NRM 67285, 19, 40.3–53.5 mm SL; Munshiganj District: Sreenagar Upazila: fish market in Shonbari; M.M. Rahman et al., 2 Dec 2014.—NRM 67286. 4, 42.3–46.5 mm SL; Munshiganj District: Sreenagar Upazila: Padma River near Sreenagar, about 23°29′16″N 90°14′11″E; M. Rahman et al., 2 Dec 2014.—NRM 67318–67319, 2, tissue; Aquarium shops in Dhaka, said to be local fish; M.M. Rahman et al., 16 May 2015.—Rangpur Division: NRM 65589, 5, 48.6–54.8 mm SL; Panchagarh District: Devigonj, Kortoa River, at Bogorbari point, under Teprigonj union, about 5 km N of Devigonj town, 26°8′15″N 88°43′8″E; M.M. Rahman, 3 May.—NRM 66730, 1, tissue, voucher in DU; Nilphamari District: Dimla, Tista barrage area, about 7 km E of Dimla Upazila, 26°10'49″N 89°2′24″E; M.M. Rahman, 2 May 2014.—Sylhet Division: NRM 68502, 4, 39.3–46.3 mm SL; NRM 68805–NRM 68806, 2, tissue; Moulovibazar District: Borolekha, Hakaluki Haor in Borolek. 24°39′42''N 92°2′5″E; M.M. Rahman et al., 26 Mar 2016.—NRM 68550, 7; 56.4–60.2 mm SL; NRM 68598, 1, tissue; Sylhet District: Jaflong, Piyain River at Jaflong. 25°11'4″N 92°0'59″'E; M.M. Rahman et al., 24 Mar 2016,—NRM 68858, 10, 47.3–54.0 mm SL; NRM 68901, 2, 49.6–52.9 mm SL; NRM 68901, 2, 49.6–52.9 mm SL; Sylhet District: Fenchuganj, Kushiyara River left bank in Fenchuganj at junction with Juri River, 24°42′19″N 91°57′16″E; M.M. Rahman et al., 22 Mar 2016.—UMMZ 208621, 8, 56.2–62.0 mm SL; Piyain Gang River at Songram Punji, ¼ mile downstream from Indian border, 25°11′N 92°1′E; W.J. Rainboth et al., 19 Feb 1978.

#### Diagnosis

*Devario devario* is a deep-bodied species (body depth at dorsal-fin origin 33–44% of SL) distinguished from all other species of *Devario* by the combination of small scales (38–50 in the lateral line, 16 circumpeduncular scales), long dorsal-fin base (iii.14½–17½), infraorbital process absent, danionine notch shallow, rostral barbel absent, maxillary barbel absent or rudimentary; supraorbital process absent; short dark P stripe posteriorly on side, extended on dorsal lobe of caudal fin; light spots or narrow irregular light and dark bars anteriorly on side.

#### Description

External aspect is shown in Fig 1. Proportional measurements and some counts are summarized in Tables 2–5. Body laterally compressed, relatively deep, with prominent chest and abdomen, more so with increasing body size; females distinctly more deep-bodied than males (33.1–37.6% SL vs. 38.3–44.3% SL; Table 2, Fig 2).

**Table 2:** Morphometry of *Devario devario* from Bangladesh. Measurements in per cents of SL, with standard deviation (SD), linear regression (a, intercept; b, slope), and correlation (Pearson’s r).

Males									Females							
	N	min	max	mean	SD	a	b	r	N	min	max	mean	SD	a	b	r
SL (mm)	6	46.8	59	51.8	5.3				11	44.7	62.0	53.6	5.9			
Body depth	**6**	**33.1**	**37.6**	**35.2**	**1.9**	-5.168	0.453	0.94	11	38.3	44.3	41.7	2.2	-9.388	0.594	0.99
Head length	6	23.4	25	24.3	0.6	2.432	0.196	0.99	11	24.2	26.7	25.1	0.7	2.308	0.208	0.98
Snout length	6	6.1	7.1	6.6	0.3	-0.35	0.196	0.92	11	6.3	7.2	6.7	0.2	0.613	0.055	0.96
Head depth	6	18	21.1	19.2	1.1	0.024	0.192	0.87	11	18.8	20.9	19.7	0.7	2.452	0.151	0.96
Head width	6	12	12.3	12.2	0.2	0.048	0.121	0.99	11	12.2	13.7	12.8	0.5	1.537	0.099	0.95
Upper jaw length	6	7.3	8.2	7.6	0.3	0.29	0.07	0.92	11	7.4	8.3	7.8	0.3	0.877	0.062	0.92
Lower jaw length	6	9.8	11.5	10.5	0.7	3.062	0.046	0.83	11	9.8	11.3	10.6	0.5	1.994	0.068	0.92
Orbital diameter	6	8.8	9.9	9.3	0.4	1.6221	0.062	0.96	11	8.8	10.5	9.5	0.6	1.845	0.061	0.86
Interorbital width	6	9.4	10.3	10	0.3	0.755	0.085	0.95	11	9.9	11.7	10.6	0.6	1.183	0.084	0.89
Caudal peduncle length	6	12.5	15.6	14.2	1.1	4.267	0.059	0.58	11	12.2	14.2	13.3	0.8	0.606	0.122	0.84
Caudal peduncle depth	6	10.7	12	11	0.5	-1715	0.144	0.96	11	11.4	12.8	12.1	0.5	-1.205	0.144	0.97
Dorsal-fin base length	6	28.2	31.5	29.8	1.3	-5.375	2.196	0.98	11	28.7	33.0	30.3	1.5	-1.808	0.338	0.92
Anal-fin base length	6	23.5	26.2	24.7	1.2	0.07	0.245	0.91	11	22.7	26.9	24.6	1.3	3.132	0.187	0.86
Predorsal length	6	49.5	52.7	51.3	1.2	5.245	0.411	0.99	11	49.5	56.8	53.9	2.0	2.456	0.585	0.96
Preanal length	6	63.5	70.2	66.6	2.8	-3.601	0.736	0.94	11	68.4	73.2	70.5	1.7	-5.143	0.802	0.99
Prepelvic length	6	46.7	50.7	49.1	1.6	-0.105	0.493	0.95	11	50.1	54.3	51.7	1.2	-0.609	0.548	0.98
Pectoral-fin length	6	20.9	22.7	21.7	0.6	-2.129	0.259	0.99	11	22.4	25.8	23.9	1.0	2.287	0.195	0.93
Pelvic-fin length	6	13.8	15.4	14.8	0.6	-2.148	0.19	0.978	11	15.0	17.2	16.3	0.7	0.915	0.145	0.92

**Table 3:** Counts of lateral line scales in species of *Devario* from Bangladesh (N = 56). Asterisk (*) marks count from holotype.

	30	31	32	33	38	41	42	43	44	45	46	47	48	50
*D*. *aequipinnatus*		4	9	7										
*D*. *anomalus*	3	1	4	4										
*D*. *coxi*		1	1*	5										
*D*. *devario*					1	1	2	3	4	2	1	1	1	1

**Table 4:** Counts of branched dorsal-fin rays in species of *Devario* from Bangladesh (N = 56). Asterisk (*) marks count from holotype.

	9½	10½	11½	12½	14½	15½	16½	17½
*D*. *aequipinnatus*	2	18	1					
*D*. *anomalus*	1	7	4					
*D*. *coxi*			5	2*				
*D*. *devario*					1	8	6	2

**Table 5:** Counts of branched anal-fin rays in species of *Devario* from Bangladesh (N = 55). Asterisk (*) marks count from holotype.

	11½	12½	13½	14½	15½	16½	17½
*D*. *aequipinnatus*	3	11	7				
*D*. *anomalus*		5	7				
*D*. *coxi*	1	1	4*	1			
*D*. *devario*					2	9	5

Predorsal area wide anteriorly, gradually more compressed posteriorly; dorsal-fin base and caudal peduncle compressed, but not acute. Predorsal contour straight or slightly curved except for slight indentation marking transverse lateralis canal. Prepelvic contour steep, straight to level of pectoral fin-base, posteriorly strongly curved with projecting chest, abdominal contour straight horizontal to about pelvic fin insertion, posteriorly horizontal or slightly ascending; chest conspicuously compressed below pectoral fin in larger specimens only; anal-fin base contour rising; ventral margin of caudal peduncle slightly concave. Snout short, rounded in lateral aspect, shorter than eye diameter. Infraorbital process absent. Supraorbital process absent. Medial margins of dentary pair straight, parallel, except for slight indentation anteriorly, representing shallow danionine notch. Mouth terminal, obliquely directed upwards. Small bony knob at dentary symphysis. Maxilla reaching to below anterior margin of orbit. Jaws equal anteriorly; lower jaw ending anteriorly at horizontal through middle of eye. Lower jaw with narrow band of about 3 rows of densely packed pointed tubercles along lateral margin. Tubercles absent from pectoral fin in all specimens. Rostral barbel absent. Maxillary barbel absent except a very short projection present on one or both sides of head in four specimens (UMMZ 208621). Supraorbital and rostral neuromast grooves present.

Lateral line complete, along 38–50 body scales (Table 3), and 2 scales on caudal-fin base; comprising one tubed scale followed by a canal running steeply caudoventrad under unperforated scales to slightly posterior to pectoral-fin base, where curved caudad and represented by scales with distinct perforation; running in a curve parallel to the ventral body outline and ending low on caudal peduncle and caudal-fin base; vertical section represented by about eight scales. Median predorsal scales 13 (1), 14 (13), 15 (3). Lateral scale rows passing between dorsal and pelvic fins ½10+1+2½ (1), ½11+1+2½ (1), ½12+1+2½ (8), ½13+1+2½ (4), ½14+1+2½ (1). Circumpeduncular scale rows 15 (1), 16 (15), 17 (1). A row of scales along anal-fin base. About ¼ of caudal-fin length scaled basally.

Dorsal- and anal-fin ray counts in Tables 4 and 5. Pectoral-fin rays i.10 (1), i.11 (13), 12 (3). Pelvic-fin rays i.7 (17). Dorsal fin inserted at highest point of dorsum, little posterior to middle of body; anterior rays gradually longer to first branched ray; distal margin straight and posterior rays gradually shorter; last ray reaching to middle of caudal peduncle. Anal fin inserted below middle rays of dorsal fin; anterior rays gradually longer to last unbranched and 1–2 anterior branched rays; distal margin slightly concave; last ray reaching to or very slightly beyond middle of caudal peduncle. Pectoral-fin insertion at about vertical through posterior margin of osseous opercle; tip pointed, not reaching to pelvic-fin origin. Pectoral-fin axial lobe well developed. Pelvic fin inserted slightly anterior to midbody, anterior to dorsal-fin origin; not reaching to anal-fin origin. Pelvic axillary scale present. Caudal fin forked, lobes round-tipped, of about equal length.

Vertebrae 17+16 = 33 (1), 17+17 = 34 (5). Pharyngeal teeth 5,4,2/2,3,5 (NRM 67285, 49.7 mm SL).

#### Colouration in preservative

Dorsum pale grey, with white or pale yellow ground colour, suffused with grey; abdomen and interstripes white or pale yellow, thin stripe in same colour close along anal-fin base and lower margin of caudal peduncle. Opercle grey or silvery depending on preservation, with several small black spots. Narrow dark brown mid-dorsal stripe from occiput to end of caudal peduncle. Cleithral spot smaller than pupil, brown or black, round or slightly deeper than wide. P stripe black, integer, about 1 scale deep, slightly expanded caudally on caudal peduncle, commencing at vertical from about first to sixth anal-fin ray, extending to end of caudal fin, covering caudal–fin rays D1–5 to their tips and rays V1–3 basally. P stripe bordered dorsally by irregular narrow light interstripe I, which anteriorly is fragmented into a series of light round or vertically extended spots. P+1 stripe dark brown, narrow, irregular, parallel with P stripe. P+2 stripe absent or indistinct, when present bordering interstripe I+1 consisting of small white spots. P-1 and P-2 stripes contained in broad dark field posterior to abdomen. I-1 interstripe short, narrow paralleling P stripe, above anal fin and forward represented by horizontal series of round or vertically extended spots which increasing in size from posterior to anterior. White spots commonly open dorsally and ventrally on middle and anterior side, giving rise to pattern of up to nine or ten narrow alternating light interbars and dark bars.

Fins hyaline, except for caudal fin as described above; dorsal-fin anterior corner with narrow black submarginal stripe and white margin.

#### Geographical distribution

*Devario devario* is reported from northern and north-eastern India, Pakistan, Bangladesh, Nepal, and Myanmar [7–8, 17, 34–37]. In Bangladesh it is found in large rivers and beels [7][35] (pers. obs.; Fig 3), contrasting with other *Devario* which occur mainly in small streams.

#### Remarks

Barbels are usually considered to be absent in *D*. *devario* (e.g., [1]), and that is the case in most specimens, but very short barbels may be present on the maxilla on one or both sides of the head [17], [present paper]. In the field *D*. *devario* is readily recognized by the very deep body and the silvery colour in life.

### Devario aequipinnatus (M’Clelland, 1839)

Figs 3 and 4

**Fig 4:**
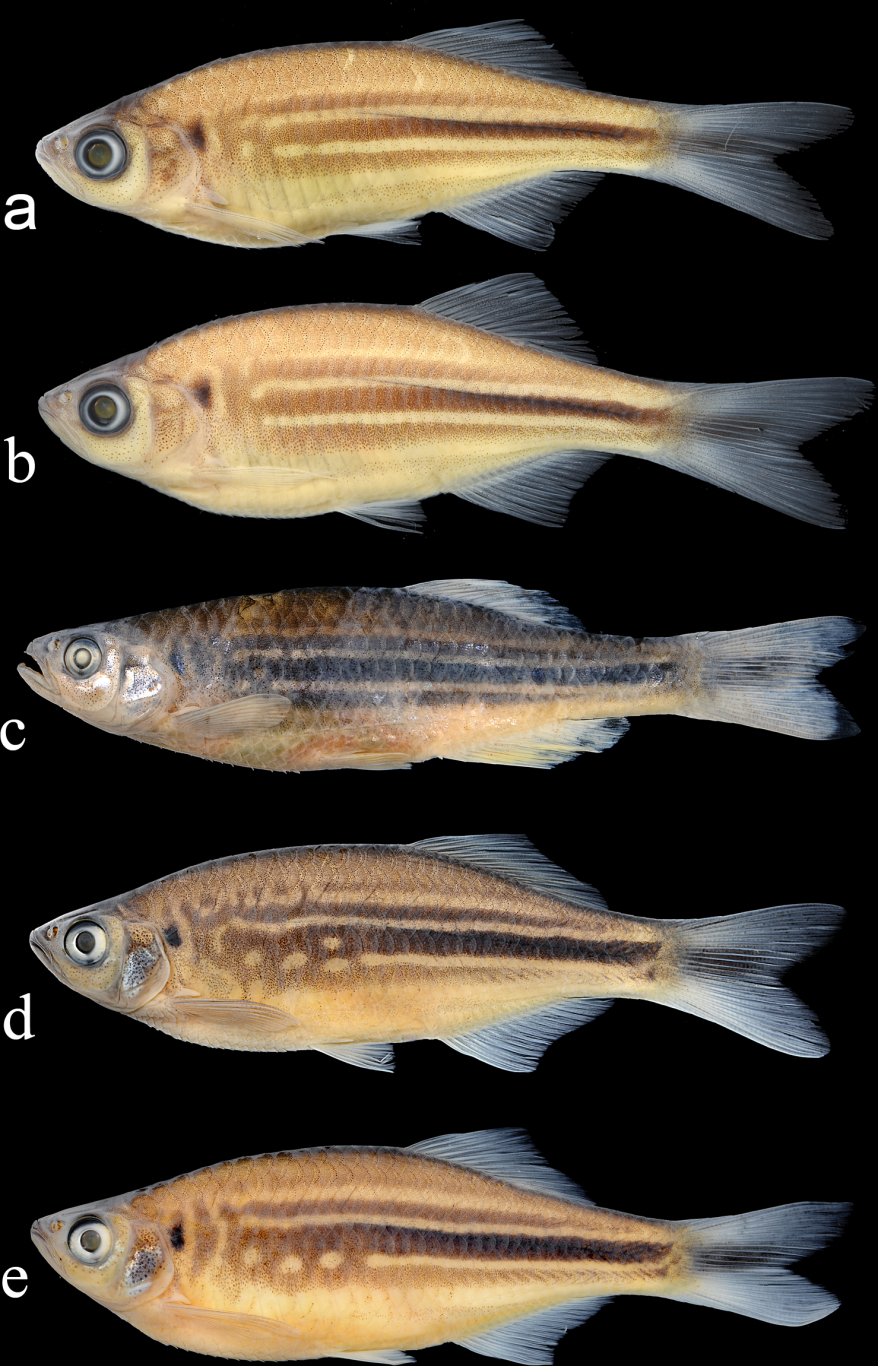
a) *Devario aequipinnatus*, young male, NRM 66216, 39.7 mm SL: Madobkundo Falls; b) *D*. *aequipinnatus*, young female, DU 9001, 42.3 mm SL: Madobkundo Falls; c) *D*. a*equipinnatus*, adult male, DU 6179, 65.7 mm SL, Khagrachori, Richong stream, preserved in 95% ethanol; d) *D*. *coxi*, holotype, adult male, DU 9002, 63.8 mm SL, Cox’s Bazar, Majerchora stream; e) *D*.*coxi*, paratype, adult female, NRM 68072, 63.4 mm SL, Cox’s Bazar, Majerchora stream.

*Perilampus aequipinnatus* M’Clelland, 1839 [13]: 393 pl. LX, Fig 1 (Assam; no extant types known)

? *Perilampus ostreographus* McClelland, 1839 [13]: 289, 392, pl. XLV, Fig 3 [= LVI, Fig 9]; throughout Assam in small indolent streams, as well as in the larger rivers,…also abundant in some parts of Bengal; no extant types known)

*Leuciscus lineolatus* Blyth, 1858 [38]: 290 (Darjiling; no extant types known)

*Devario cyanotaenia* Bleeker, 1860 [33]: 860:283 (name only; reference to pl. 56 [LVI in original], Fig 9 in M’Clelland, 1839 [13], which corresponds to legend for pl. XLV, Fig 3 on p. 314; unnecessary replacement name for *Perilampus ostreographus* McClelland, 1839).

? *Danio assamensis* Barman, 1984 [18]: 163 (holotype SZI FF1861; type locality Streamlets round about Tangla, Darrang dist., Assam).

### Material examined

#### All from Bangladesh

*Sylhet Division*: Meghna River drainage:DU 9001, 1, 42.3 mm SL; DU 0201091 and NRM 66705 (fin clip), 1, 37.6 mm SL; NRM 66216, 8, 34.3–49.0 mm SL; Moulovibazar District: Meghna River drainage: Madobkundo waterfall, hill streams about 5 km from Dakshinbagh railway station on the Kulaura-Shabajpur track, 24°33′17.0″N 92°13'26.4″E; M.M.Rahman, 26 Apr 2014.—NRM 66258 (T9865) 1, 60.2 mm SL; NRM 66259 (T9866) 1, 53.7 mm SL; Moulovibazar District: Madobkundo waterfall, aquarium specimen from the wild, kept in aquarium; M.M. Rahman.—*Chittagong Division*: Feni River drainage: DU 6104, 2, 33.7–48.1 mm SL; Feni River and Shitakunda hillstream, Mohamaya hillstream; M.M. Rahman, 31 May 2015.—DU 6179, 65.7 mm SL; DU 6180, 3, 35.1–50.4 mm SL; DU 6244, 1, 60.9 mm SL; Khagrachori Sadar: Richong stream; M.M. Rahman, 25 Apr 2015.—DU 6184, 3, 29.7–36.9 mm SL; Khagrachori Sadadar: Richong stream at Richong waterfall, 23°03′57″N, 91°56′38″E; M.M. Rahman, 25 Apr 2015.—DU 6188, 3, 21.5–33.9 mm SL; Khagrachori Sadar: Bangmara stream beside Chittagong–Khagrachori highway, 23°03′50″N, 91°54′18″E; M.M. Rahman, 24 Apr 2015.—DU 6253, 1, 47.8 mm SL; Feni River and Shitakunda hill stream, Mohamaya Hill, 22°29′05″N, 091°21′07″E; M.M. Rahman, 29 May 2015—UMMZ 209008, 4, 34.2–47.0 mm SL; Koilla Khal, 6 miles E of Feni–Chittagong Highway on road to Ramgarh, 22°55′N, 91°36′E; W.J. Rainboth et al., 3 Feb 1978.—Karnafuli River drainage: CAS 94526. 8, 35.3–54.5 mm SL.—Karnafuli River drainage: small stream 7 km E of Khagrachari town; T.R. Roberts, 7 Jun 1996.—NRM 66813 (T10223), 1, 38.5 mm SL; hill stream at northwestern margin of Chittagong University campus, in and below rapids, 2°28″26′N, 91°46’59″E; M.M. Rahman et al., 29 Nov 2014—NRM 66832 (T10198) 1, 54.1 mm SL; Bangchora hill stream in Kaptai, near hydropower station, 22°29′45″N, 92°11′12″E; M.M. Rahman et al., 28 Nov 2014.

#### Diagnosis

*Devario aequipinnatus* is distinguished from all other *Devario* by the particular colour pattern: presence of distinct, straight P+1, P, and P-1 stripes, of which the P stripe is wider than the others, equal in width from anterior to posterior end, or slightly tapering caudad; anterior part of Interstripe I (below P stripe) may be interrupted at one or two points anteriorly. Similar to *D*. *coxi* in colour pattern, with distinct P+1, P and P stripes along the side, but P and P-1 integer to close to pectoral girdle, and interstripe I integer except occasional interruption anteriorly (vs. stripes confluent anteriorly and containing light spots). Distinguished from *D*. *coxi* also by the combination of less dorsal-fin rays (9½–10½, exceptionally 11½ vs. 11½–12½), circumpeduncular scales (12, rarely 14, vs. 14); slightly more slender body (body depth 26.7–30.8 vs. 29.9–33.2% SL), and longer upper jaw (upper jaw length 9.2–12.0 vs. 8.3–9.3% SL). Distinguished further from striped species of *Devario* in southern and south-western India, *D*. *fraseri*, *D*. *malabaricus*, *D*. *mysoricus*, and *D*. *neilgherriensis* by presence vs. absence of infraorbital process.

#### Description

External aspect is shown in Fig 4A and 4B. Proportional measurements and some counts are summarized in Tables 3–6.

**Table 6:** *Devario aequipinnatus* from Bangladesh. Measurements in per cents of SL, with standard deviation (SD); linear regression (a, intercept; b, slope), and correlation (Pearson’s r) if significant in ANOVA at p = 0.

	N	min	max	mean	SD	A	B	r
SL (mm)	21	34.3	65.7	46.3	7.9			
Body depth	21	26.7	30.8	28.6	1.2	0.987	0.264	0.97
Head length	21	22.9	29.2	25.7	1.7	3.324	0.183	0.95
Snout length	21	6.9	8.3	7.6	0.4	0.629	0.061	0.97
Head depth	21	16.3	20.4	18.2	1.3	2.127	0.135	0.91
Head width	21	11.7	14.6	13.1	0.7	1.255	0.103	0.94
Upper jaw length	21	9.2	12.0	10.4	0.8	1.646	0.068	0.93
Lower jaw length	21	11.1	14.9	13.1	1.1	2.743	0.071	0.89
Orbital diameter	21	6.7	10.2	8.6	0.9	2.353	0.034	0.85
Interorbital width	21	9.4	11.6	10.6	0.6	1.430	0.074	0.96
Caudal peduncle length	21	17.0	21.3	19.0	1.3	-1.165	0.216	0.96
Caudal peduncle depth	21	10.7	12.4	11.9	0.5	0.049	0.120	0.97
Dorsal-fin base length	21	15.7	20.2	18.4	1.2	1.067	0.160	0.92
Anal-fin base length	21	16.1	21.3	18.9	1.2	0.122	0.186	0.93
Predorsal length	21	54.7	64.0	58.7	2.0	2.357	0.534	0.98
Preanal length	21	63.0	70.0	65.1	1.7	0.298	0.644	0.99
Prepelvic length	21	44.1	50.0	47.8	1.6	1.133	0.453	0.98
Pectoral-fin length	21	19.5	24.5	22.1	1.5	3.399	0.146	0.94
Pelvic-fin length	21	12.2	16.3	14.4	1.2	1.736	0.106	0.89
Rostral barbel length	20	2.6	6.7	4.5	1.2			
Maxillary barbel length	21	0.6	2.8	1.5	0.5			

Body laterally compressed, elongate; no sexual dimorphism in body shape. Predorsal area wide and flat anteriorly, gradually more compressed, dorsum acute under dorsal-fin base, postdorsally slightly wider, gradually more compressed to caudal-fin base. Predorsal contour straight ascending or ascending slightly concave anteriorly, raised gradually from transverse lateralis canal; slightly curved to dorsal-fin base; from dorsal-fin base posteriorly to base of caudal fin straight sloping. Prepelvic contour sloping, straight or slightly concave beneath pectoral-fin base, posteriorly nearly horizontal, slightly curved; chest not conspicuously compressed below pectoral fin; anal-fin base contour rising; ventral margin of caudal peduncle about straight, horizontal. Snout short, rounded in dorsal aspect, subtriangular in lateral aspect, about as long as eye diameter. Infraorbital process flat, wider than high or square, width about half pupil diameter, with truncate or slightly rounded distal margin. Supraorbital process absent. Danionine notch caudally margined by well-developed anteromediad projecting blunt laminar dentary process. Skin cover absent from distal part of infraorbital process and anterior margin of supraorbital. Mouth terminal, obliquely directed upwards. Small bony knob at dentary symphysis. Maxilla reaching to below anterior margin of orbit. Jaws equal anteriorly; lower jaw ending anteriorly at horizontal through middle of eye. Upper lip anteriorly much wider than lower lip, which not much wider than barbel. Lower jaw with lateral row of about 25 sharp, short, conical tubercles, followed medially by about six more parallel rows, forming wide band of tubercles extending to the medial margin of the dentary. In males rows of strong, densely arranged sharp-tipped conical tubercles on anterior 5 branched rays of pectoral fin; pectoral-fin tubercles absent in females. Rostral barbels short, length of infraorbital 1 or to base of maxillary barbel or slightly longer; maxillary barbel much shorter, at most half length of rostral barbel. Supraorbital and rostral neuromast grooves prominent.

Lateral line complete, along 31–33 trunk scales (Table 3), and 2 scales on caudal-fin base; comprising one tubed scale followed by a canal running steeply caudoventrad under unperforated scales to slightly posterior to pectoral-fin base, where curved caudad and represented by scales with distinct perforation; running in a curve parallel to the ventral body outline and ending low on caudal peduncle and caudal-fin base; vertical section represented by about six scales. Median predorsal scales 14 (15), 15 (5). Lateral scale rows passing between dorsal and pelvic fins ½6+1+2½ (6), ½7+1+2½ (12). Circumpeduncular scale rows 12 (21), 14 (4). A row of scales along anal-fin base. About ¼ of caudal-fin length scaled basally.

Dorsal- and anal-fin ray counts in Tables 4 and 5. Pectoral-fin rays i.10 (5), i.11 (6), i.12 (8), i.13 (1). Pelvic-fin rays i.7 (20). Dorsal fin inserted at highest point of dorsum, little posterior to middle of body; anterior rays gradually longer to last unbranched and 1–2 anterior branched rays; distal margin straight and posterior rays gradually shorter; last ray reaching to middle of caudal peduncle. Anal fin inserted below anterior rays of dorsal fin; anterior rays gradually longer to last unbranched and 1–2 anterior branched rays; distal margin slightly concave; last ray reaching to or very slightly beyond middle of caudal peduncle. Pectoral-fin insertion at about vertical through posterior margin of osseous opercle; tip rounded, extending to pelvic-fin origin. Pectoral-fin axial lobe well developed. Pelvic fin inserted slightly anterior to mid-body, anterior to dorsal-fin origin; not reaching to anal-fin origin. Pelvic axillary scale present. Caudal fin forked, lobes round-tipped, of about equal length, but lower very slightly longer and broader.

Vertebrae 16+18 = 34 (1), 17+17 = 34 (5), 17+18 = 35 (1), 18+17 = 35 (1). Pharyngeal teeth 5,4,1/1,4,5 (NRM 66216, 45.5 mm SL).

#### Colouration in preservative

Ground colour yellowish white. Sides of head with sparse pigmentation. Dorsum pale grey. Narrow black dorsal midline from occiput to end of caudal peduncle. P stripe about one scale wide, slightly decreasing in width caudally, margins even; dark brown, darker caudally, ending anteriorly 2–3 scales posterior to cleithrum, like other horizontal stripes and interstripes, posteriorly at end of caudal peduncle. Anterior to P stripe diffuse brownish, followed by contrasting yellowish white short vertical bar and wedge shaped black or dark brown vertically oriented cleithral spot. Interstripe I less than one scale wide, paralleling P stripe, anteriorly distinctly narrower than P stripe, posteriorly relatively less distinctly narrower. Interstripe I+1 Paralleling P stripe, anteriorly very narrow, slightly wider caudally, still less than 1 scale wide. P+1 stripe paralleling P stripe, less than one scale wide, pale brown, anteriorly on caudal peduncle fading into pale grey colour of dorsum. Interstripe I+2 short, parallel to, and much narrower than P+1 stripe. P-1stripe brown narrower than P stripe and gradually narrower caudally, ending on caudal-fin base. Interstripe I paralleling P-1 stripe, narrower than P-1 stripe and narrower caudally. P-2 stripe indistinct, formed by scattered melanophores from above posterior anal-fin base anteriorly to anterior level of other stripes and interstripes. In some specimens, one or two light spots anteriorly in P stripe, or I-1 stripe anteriorly partly discontinuous. Pigmentation of P and P-1 stripes continuous anteriorly. Caudal fin hyaline except dark grey indistinct band on lower rays of upper lobe. Dorsal fin dusky basally, corner and distal margin hyaline. Remaining fins hyaline. Specimens in ethanol grey where formalin preserved specimens brown. Cleithral spot variable in shape, wedge-shaped or slightly oval. P stripe of almost equal width in the larger specimens, and also with variable pattern of short darker stripes and light spots at anterior end of dark stripes.

#### Geographical distribution

In Bangladesh, *D*. *aequipinnatus* is found in small numbers in fast flowing small streams within the Karnafuli, Feni, and Meghna drainages (Fig 3). Records from the Tista and Brahmaputra drainages in India suggest that it occurs in similar habitats further to the west or northwest. *Devario aequipinnatus* was reported from Bangladesh by [11], [35]. It was given a wide range in northern India, Nepal, Bangladesh, Pakistan, Sri Lanka, Thailand and China by Barman [17], and similar wide distribution by other authors (e.g., [36]). In Bangladesh the species appears to be restricted to small forest hill streams.

#### Remarks

Based on the comparative material used herein, *D*. *aequipinnatus* is present outside Bangladesh in the upper Brahmaputra, Barak, and Tista Rivers in India and the upper Khosi River, Ganga drainage, in Nepal, and has probably a wider distribution in northeastern and northern India, and adjacent Nepal. In the Indian comparative material meristics are: branched dorsal fin rays 9½ (4), 10½ (14), 11½ (6), 12½ (1); lateral line scales 30 (1), 31 (2), 32 (2), 33 (8), 34 (1), 35 (1); circumpeduncular scales 12 (25).

### *Devario coxi*, new species

Figs 3, 4B and 4C

urn:lsid:zoobank.org:act:2B4E4267-A398-4101-9D76-8E3A50AAB7AA.

#### Holotype

DU 9002, adult male, 63.8 mm SL; Bangladesh, Chittagong Division, Cox’s Bazar District, Majerchora stream, 10 km south of Cox's Bazar, 21°23′45″N; M.M. Rahman et al., 8 May 2015.

#### Paratypes

All from Bangladesh, Chittagong Division. CAS 94524, 2, 65.5–72.1 mm SL; small foothill tributary of Matamuhuri River across Matamuhuri River from Alikadam town (about 21°40′N,92°18′E); T.R. Roberts, 4 Jun 1996.—DU 9003, 1, 71.6 mm SL; NRM 67379 (T10544) 1, 62.6 mm SL; NRM 68072, 2, 63.4–66.6 mm SL; Same data as holotype.

#### Diagnosis

Similar to *D*. *aequipinnatus* in colour pattern, with distinct P+1, P and P stripes along the side, but P and P-1 stipes confluent anteriorly and containing light spots (vs. integer to close to pectoral girdle, and interstripe I integer except occasional interruption anteriorly). Distinguished from *D*. *aequipinnatus* also by the combination of more branched dorsal-fin rays (11½–12½ vs. 9½–10½, exceptionally 11½), more circumpeduncular scales (14, vs. 12, rarely 14); slightly deeper body (body depth 29.9–33.2 vs. 26.7–30.8% SL), and shorter upper jaw (upper jaw length 8.3–9.3 vs. 9.2–12.0% (SL). Distinguished further from striped species of *Devario* in southern and south-western India, *D*. *fraseri*, *D*. *malabaricus*, *D*. *mysoricus*, and *D*. *neilgherriensis* by presence vs. absence of infraorbital process.

#### Description

External aspect is shown in Fig 4C and 4D. Some meritic data is presented in Tables 3–5; proportional measurements are summarized in Table 7. Counts from holotype marked with asterisk.

**Table 7:** Morphometry in *Devario coxi*. Measurements in per cents of SL, with standard deviation (SD); linear regression (a, intercept; b, slope), and correlation (Pearson’s r) if significant in ANOVA at p = 0. HT = separate data for holotype.

	HT	N	min	max	mean	SD	a	b	r
SL (mm)	63.8	7	62.6	71.6	66.4	3.7			
Body depth	29.9	7	29.9	33.2	30.9	1.3	-2.178	0.355	0.95
Head length	23.4	7	22.8	24.0	23.4	0.4	0.253	0.240	0.99
Snout length	6.9	7	6.6	8.0	7.0	0.5	-0.370	0.077	0.98
Head depth	16.8	7	15.9	18.7	16.9	0.9	0.058	0.176	0.96
Head width	12.4	7	12.0	13.1	12.5	0.4	0.203	0.128	0.99
Upper jaw length	9.2	7	8.3	9.3	8.9	0.4	-0.019	0.096	0.96
Lower jaw length	12.2	7	11.0	13.1	12.0	0.7	-0.032	0.118	0.97
Orbital diameter	8.0	7	7.3	8.0	7.7	0.3	0.490	0.071	0.95
Interorbital width	9.7	7	9.3	10.3	9.9	0.3	0.084	0.105	0.98
Caudal peduncle length	17.1	7	17.1	19.2	18.1	0.9	1.081	0.151	0.93
Caudal peduncle depth	12.4	7	11.1	12.9	12.0	0.6	-0.233	0.131	0.99
Dorsal-fin base length	21.0	7	19.3	21.9	20.5	1.0	-3.538	0.262	0.91
Anal-fin base length	20.2	7	18.1	21.4	19.3	1.1	-0.915	0.227	0.96
Predorsal length	59.6	7	56.5	59.8	58.3	1.1	1.076	0.566	0.99
Preanal length	64.9	7	64.4	69.5	66.5	1.9	-1.076	0.680	0.99
Prepelvic length	45.8	7	45.8	50.6	48.0	1.8	-0.206	0.485	0.99
Pectoral-fin length	21.6	7	20.8	22.6	21.8	0.7	0.326	0.232	0.95
Pelvic-fin length	14.7	7	14.2	15.6	14.8	0.4	-0.352	0.169	0.95
Rostral barbel length	3.6	6	2.5	4.1	3.6	0.6			
Maxillary barbel length	1.3	7	0.4	1.7	0.9	0.4			

Body laterally compressed, elongate, females slightly more deep-bodied than males (30.2–33.2% SL vs. 27.8–39.0%), and with greater preanal distance (66.0–69.5% SL vs. 63.6–64.9%). Predorsal area wide and flat anteriorly, gradually more compressed, dorsum acute under dorsal fin base, postdorsally slightly wider, gradually more compressed to caudal fin base. Predorsal contour in small specimens straight ascending; in large adults ascending slightly concave anteriorly, raising gradually from transverse lateralis canal, slightly curved to dorsal-fin base; from dorsal-fin base posteriorly to base of caudal fin straight sloping. Prepelvic contour sloping, straight or slightly concave beneath pectoral-fin base, posteriorly nearly horizontal, slightly curved, more so in females; chest not conspicuously compressed below pectoral fin; anal-fin base contour rising; ventral margin of caudal peduncle about straight, horizontal. Snout short, rounded in dorsal aspect, subtriangular in lateral aspect, about as long as eye diameter. Infraorbital process flat, wider than high, width about half pupil diameter, with truncate or slightly rounded distal margin. Supraorbital process absent. Danionine notch caudally margined by well-developed anteromediad projecting, blunt laminar dentary process. Skin cover absent from distal part of infraorbital process, dentary process, and anterior margin of supraorbital. Mouth terminal, obliquely directed upwards. Small bony knob at dentary symphysis. Maxilla reaching to below anterior margin of orbit. Jaws equal anteriorly; lower jaw ending anteriorly at horizontal through middle of eye. Upper lip anteriorly much wider than lower lip, which not much wider than barbel. Lower jaw with lateral row of about 20–25 short sharp, short, conical tubercles, followed medially by one or more parallel rows, or a wider band of tubercles extending to the medial margin of the dentary; except for lateral row, tuberculation variably developed, from distinct to rudimentary without obvious correlation with sex. In males rows of strong, densely arranged sharp-tipped conical tubercles on anterior six branched rays of pectoral fin; pectoral-fin tubercles absent in females. Rostral barbels short, length of infraorbital 1 or to base of maxillary barbel; maxillary barbel much shorter, at most half length of rostral barbel. Supraorbital and rostral neuromast grooves prominent.

Lateral line complete, along 31–33 trunk scales (Table 3), and 2 scales on caudal-fin base; comprising one tubed scale followed by a canal running steeply caudoventrad under unperforated scales to slightly posterior to pectoral-fin base, where curved caudad and represented by scales with distinct perforation; running in a curve parallel to the ventral body outline and ending low on caudal peduncle and caudal-fin base; vertical section represented by about six scales. Median predorsal scales 14 (5), 15* (2). Lateral scale rows passing between dorsal and pelvic fins ½6+1+2½ (6), ½7+1+2½* (2). Circumpeduncular scale rows 14* (7). A row of scales along anal-fin base. About ¼ of caudal-fin length scaled basally.

Dorsal- and anal-fin ray counts in Tables 4 and 5. Pectoral-fin rays i.11 (5), i.12* (2). Pelvic-fin rays i.7* (7). Dorsal fin inserted at highest point of dorsum, little posterior to middle of body; anterior rays gradually longer to last unbranched and 1–2 anterior branched rays; distal margin straight and posterior rays gradually shorter; last ray reaching to middle of caudal peduncle. Anal fin inserted below anterior rays of dorsal fin; anterior rays gradually longer to last unbranched and 1–2 anterior branched rays; distal margin slightly concave; last ray reaching to or very slightly beyond middle of caudal peduncle. Pectoral-fin insertion at about vertical through posterior margin of osseous opercle; tip rounded, extending to pelvic-fin origin. Pectoral-fin axial lobe well developed. Pelvic fin inserted slightly anterior to midbody, anterior to dorsal-fin origin; not reaching to anal-fin origin. Pelvic axillary scale present. Caudal fin forked, lobes round-tipped, of about equal length, but lower very slightly longer and broader.

Vertebrae 17+18 = 35 (3), 18+17 = 35 (2). Pharyngeal teeth 5,4,2/2,4,5 (DU 9003, 71.6 mm SL).

#### Coluration in preservative

Ground colour yellowish white. Dorsum pale brownish. Narrow black dorsal midline from occiput to end of caudal peduncle. P stripe about one scale wide, of uniform width throughout, margins even; greyish brown anteriorly, darker caudad, dark brown to black posterior to trunk middle; ending at end of caudal peduncle; 1–3 small light spots may be present close to anterior end of stripe. Interstripe I anteriorly replaced with three light spots. Greyish brown pigmentation in P and P-1 stripes continuous anterior to trunk middle. P+1 stripe slightly lighter than and one half as wide as P stripe, ending fading anteriorly on caudal peduncle. P+2 stripe not present or narrower than P+1 stripe, ending below end of dorsal fin base. P-2 stripe along abdominal side, faint, barely visible. P-1 stripe as wide as P stripe and light greyish brown anteriorly; indistinct lower border; gradually narrower posteriorly reduced to thin line on caudal peduncle. Interstripes I and I+1 each about half as wide as P stripe, straight, uniformly light, Interstripe I+2 slightly wider han P+1, ending diffusely anteriorly on caudal peduncle. Black, vertically oriented cleithral spot covering part of first lateral-line scale; posterior to it two greyish brown bars, slightly curved (concave rostrally), from anterior end of P+1 stripe to anterior end of P-1 stripe. Dark brown dot on caudal-fin base at end of P and P-1 stripes. Caudal fin hyaline except dark grey indistinct band on middle rays. Dorsal fin with scattered melanophores along middle; and white anterior corner and distal margin. Anal fin with scattered melanophores and white anterior corner. Pectoral and pelvic fins hyaline.

#### Live colouration

Adults observed in the field with dorsum steel blue, P+1 stripe green, P and P-1 stripes pale blue or blue-green, abdomen and chest white. Light interstripes yellow. Middle rays of caudal fin grey-green, lobes red.

#### Geographical distribution

Known only from a small stream near Cox’s Bazar, and the lower Matamuhuri River in Bangladesh (Fig 3).

#### Etymology

The specific name is a noun in the genitive case, referring to the type locality area (Cox’s Bazar), but could also be understood as referring to the gene fragment used to identify the species (*cytochrome c oxidase subunit 1*, often shortened to COXI); or to Hiram Cox (1760–1799), after whom Cox’s Bazar was named.

#### Remarks

Only two specimens were available for sequencing; remaining specimens referred to this population were identified by the unique combination of 14 circumpeduncular scales and 11½-12½ unbranched dorsal-fin rays.

### *Devario anomalus* Conway, Mayden & Tang, 2009

Figs 3, 5 and 6

**Fig 5:**
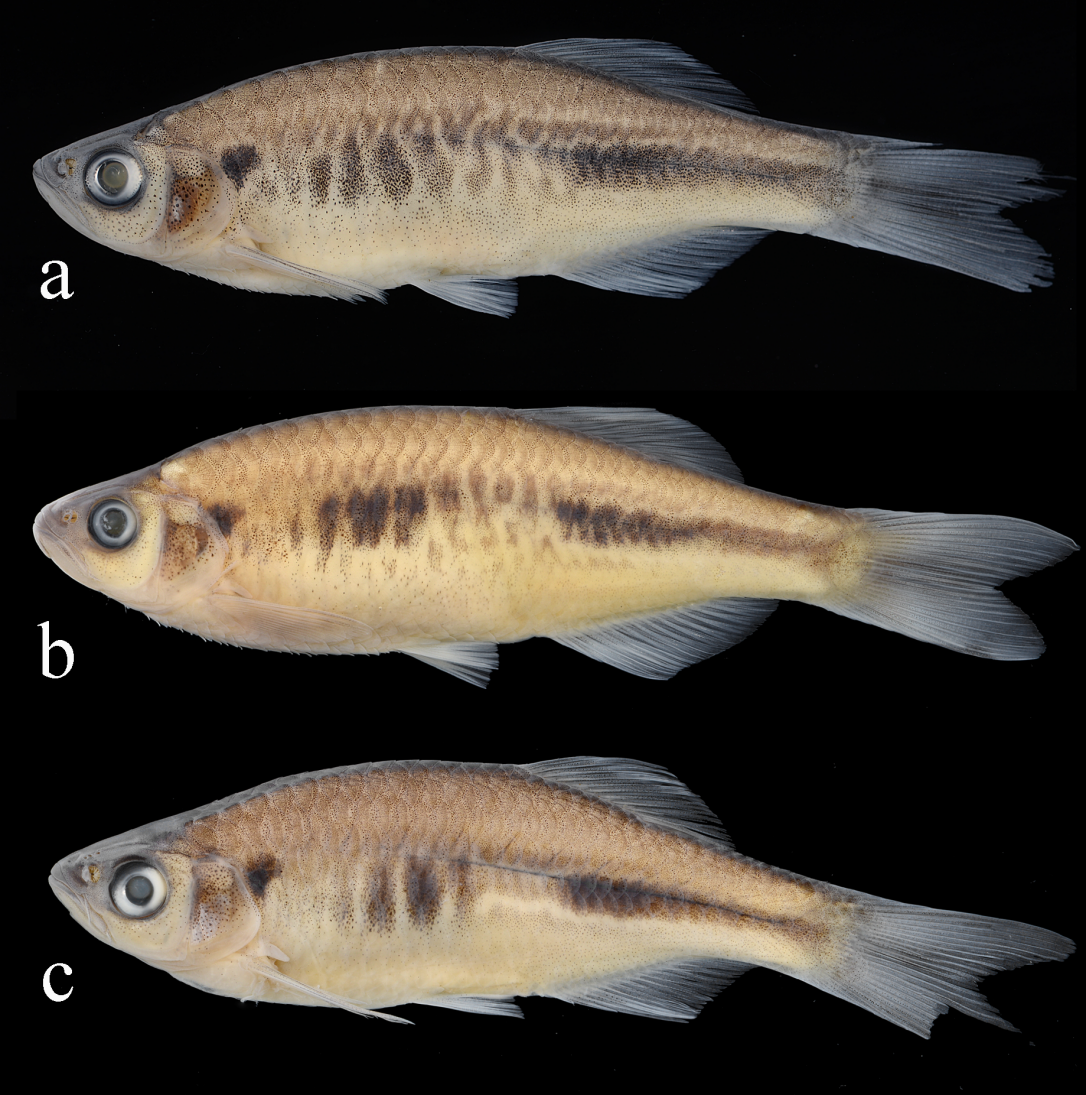
*Devario anomalus*. a) young male, NRM 68075, 50.8 mm SL, Cox’s Bazar, Barachora Stream; b) adult female, 65574, 66.2 mm SL, Cox’s Bazar, Barachora stream; c) young male, NRM 68186, 45.1 mm SL, Bandarban, Sangu River near Ruma.

**Fig 6:**
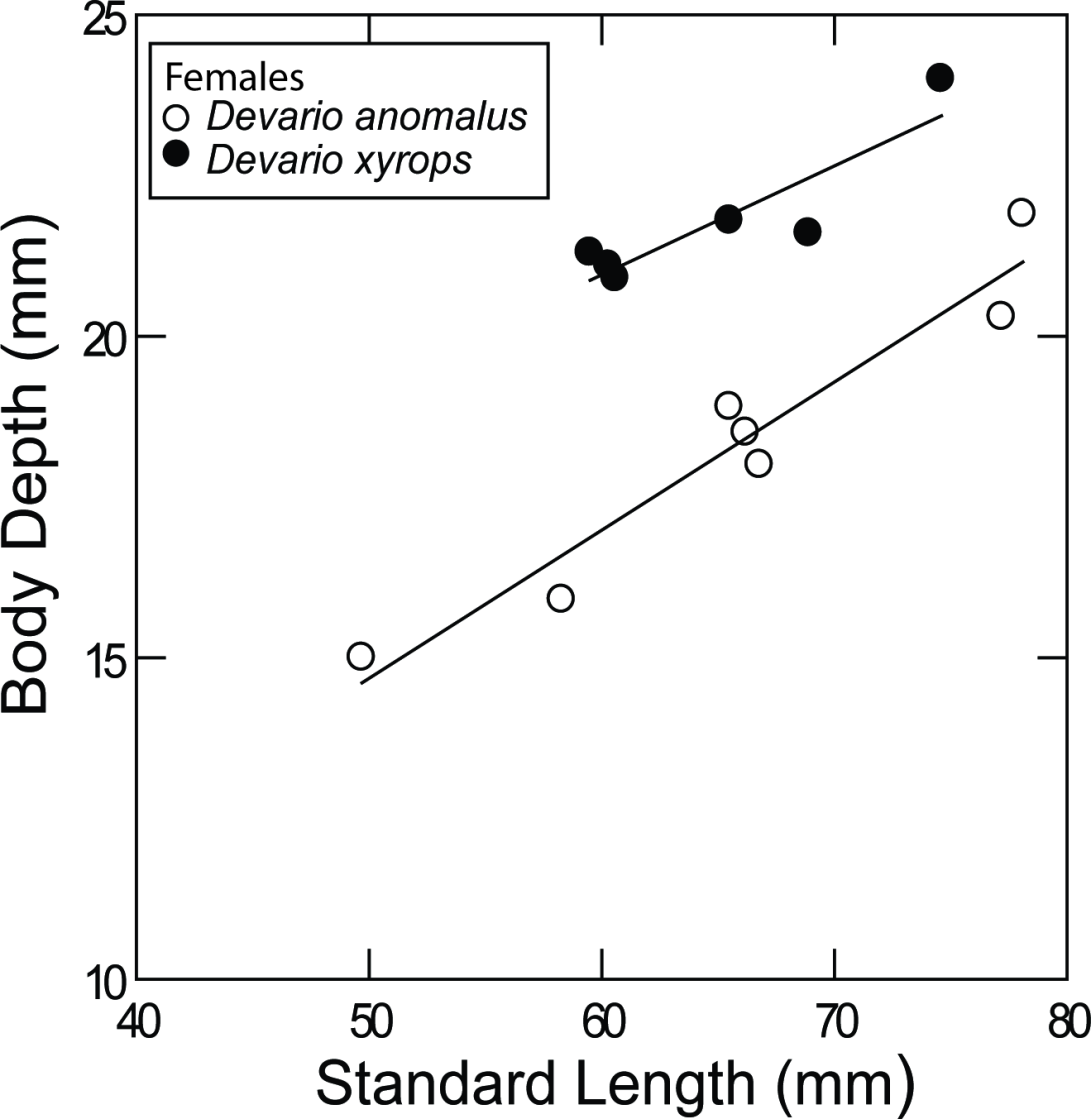
Difference in body depth in females of *Devario anomalus* and *D*. *xyrops*.

*Devario anomalus* Conway et al., 2009 [9]: 50, Fig 1. (Holotype UF 174139. Type locality: Bangladesh: Chittagong Division, stream crossing highway at market southeast of Cox’s Bazar, 21°21′18.4″N, 92°1′29.90″E.)

#### Material examined

All from Bangladesh, Chittagong Division: *Cox’s Bazar District*: *coastal streams south of Cox’s Bazar*: NRM 65562(T9874), 1, 73.1 mm SL; No locality data.—NRM 65574, 7, 53.5–77.2 mm SL; NRM 66501 (T9995); 1, tissue; Barachara hillstreams about 6 km SE of Cox’s Bazar town, 21°23′46″N 92°0′2″E; A.R. Mollah, 4 Apr 2014.—NRM 67333, 1, 67.2 mm SL; Kudung Cave in Teknaf Game Reserve, 21°5′32″N 92°10′11″E; M.M. Rahman, et al., 9 May 2015 (SRL-2015-004).—NRM 67810(T10825) 1, 43.0 mm SL; 67811(T11339), 1, 56.7 mm SL; 68058, 2, 36.5–42.3 mm SL; Donumia Stream, 10 km north of Teknaf town, 70 km south of Cox's Bazar, 20°55′24″N 92°15′47″E. M.M. Rahman et al. 9 May 2015 (SRL-2015-012).—NRM 68075, 20, 19.4–78.1 mm SL; 68081, 23, 21.4–32.5 mm SL; NRM 67398 (T10661), NRM 67399 (T10659), NRM 67400(T10728), NRM 67401(T10762), NRM 67403 (T10748), 6, 26–30.3 mm SL; Barachora stream, 4 km S of Cox’s Bazar, 21°23′45″N 92°0′13″E; M.M. Rahman et al., 8 May 2015 (SRL-2015-001).—NRM 68131, 2, 79.2–96.7 mm SL; Cox’s Bazar; M.M. Rahman, 3 Dec 2012 (MMR031212). *Bandarban District*, *Sangu River drainage*: CAS 94595, 2, 33.5–58.5 mm SL; small sandy hillstream near Ramail [not located], E of Bandarban; T.R. Roberts, 20 May 1996.—NRM 67936 (T10786), 1, 55.0 mm SL; NRM 67937 (T10787) 1, 65.5 mm SL; NRM 68186, 1, 45.1 mm SL; Ruma, Mrong Bazar, small stream tributary to Sangu River, below bridge, 22°3′27″N 92°18′56″E; M.M. Rahman et *al*., 14 May 2015 (SRL-2015-026).

#### Diagnosis

*Devario anomalus* shares uniquely with *D*. *xyrops* an interrupted P stripe; the anterior portion is represented by several short vertical bars, the posterior portion by a dark stripe tapering caudally, and not continued onto the caudal fin. Narrow P+1 and P-1 stripes may be present posteriorly on the side. Distinguished from *D*. *xyrops* by more slender body, 26.3–30.2% SL vs. 31.4–35.8% SL in females (Fig 6)

#### Description

External aspect is shown in Fig 5. Some meristics is presented in Tables 3–5; proportional measurements are summarized in Table 8.

**Table 8:** Morphometry of *Devario anomalus*. Measurements in per cents of SL, with standard deviation (SD); linear regression (a, intercept; b, slope), and correlation (Pearson’s r) if significant in ANOVA at p >0.9 but not shown for males in which not significant for most measurements.

Males						Females							
	N	min	max	mean	SD	N	min	max	mean	SD	a	b	r
SL (mm)	5	50.8	60.0	55.5	3.6	7	49.7	78.1	66.0	10.0			
Body depth	5	25.9	28.5	27.2	1.0	7	26.3	30.2	27.9	1.3	3.155	0.230	0.89
Head length	5	24.0	26.0	24.8	0.8	7	24.2	26.0	24.8	0.7	-1.102	0.265	0.90
Snout length	5	6.8	8.0	7.4	0.5	7	7.3	7.8	7.5	0.2	-0.680	0.085	0.39
Head depth	5	15.7	17.3	16.5	0.7	7	15.4	17.5	16.6	0.8	-0.791	0.179	0.78
Head width	5	12.4	13.2	12.9	0.3	7	12.2	13.6	13.0	0.5	-0.994	0.146	0.94
Upper jaw length	5	10.2	11.6	10.8	0.6	7	10.1	11.5	10.6	0.5	1.089	0.089	0.62
Lower jaw length	5	12.8	14.2	13.4	0.6	7	11.7	13.9	12.7	0.8	2.939	0.081	0.78
Orbital diameter	5	7.1	7.9	7.4	0.3	7	6.9	7.7	7.1	0.3	0.535	0.062	0.79
Interorbital width	5	9.8	10.4	10.1	0.2	7	9.6	10.5	10.2	0.3	-0.195	0.105	0.96
Caudal peduncle length	5	17.5	19.3	18.6	0.7	7	17.3	19.0	18.3	0.6	-0.381	0.189	0.80
Caudal peduncle depth	5	11.9	12.8	12.3	0.4	7	11.5	12.5	12.0	0.4	-0.099	0.122	0.97
Dorsal-fin base length	5	18.5	20.2	19.3	0.6	7	17.7	19.6	18.6	0.7	0.272	0.175	0.98
Anal-fin base length	5	19.1	22.3	20.2	1.3	7	19.2	21.1	20.4	0.7	0.612	0.194	0.97
Predorsal length	5	54.9	61.8	59.0	2.6	7	55.7	62.0	60.1	2.1	0.942	0.586	0.83
Preanal length	5	62.9	65.6	64.0	1.0	7	63.8	66.2	64.7	0.8	2.080	0.615	0.99
Prepelvic length	5	44.0	47.4	46.1	1.4	7	44.9	47.8	46.4	1.1	2.007	0.433	0.95
Pectoral-fin length	5	20.2	22.0	21.1	0.7	7	20.5	23.3	21.8	0.9	2.837	0.674	0.89
Pelvic-fin length	5	14.1	16.0	14.7	0.7	7	13.2	15.7	14.1	0.8	2.498	0.423	0.94
Rostral barbel length	5	4.5	6.2	5.3	0.7	7	3.8	6.0	4.9	0.7			
Maxillary barbel length	5	1.7	2.6	1.9	0.4	7	1.7	2.4	2.1	0.3			

Body laterally compressed, elongate. Predorsal contour straight or slightly curved, ascending, with or without slight indentation marking position of transverse lateralis canal; sloping posteriorly from dorsal-fin insertion. Prepelvic contour curved, more so in females; chest more compressed below pectoral fin, but not keeled. Snout short, rounded in dorsal aspect, subtriangular in lateral aspect, about as long as eye diameter in lateral aspect. Infraorbital process laminar, pointed or broader than high with truncate distal margin. Danionine notch caudally margined by blunt anteromediad projecting, laminar dentary process. Skin cover absent from distal part of infraorbital process, dentary process, and anterior margin of supraorbital. Mouth terminal, obliquely directed upwards. Small bony knob at dentary symphysis. Maxilla reaching to below anterior margin of orbit. Jaws equal anteriorly; lower jaw ending anteriorly at horizontal through middle of eye, posteriorly at vertical through middle of eye. Lower jaw with large conical tubercles in several rows in juveniles (including smallest specimen, 19.4 mm SL); tubercles gradually reduced in relative height with increasing body size; in adults lower jaw with 2–3 rows of minute conical tubercles concentrated on lateral surface, occasionally absent, in large specimens a few tubercles along margins of danionine notch; tuberculation in adults variably developed from hardly visible to well-developed without correlation with sex. Seven specimens, 50.8–79.2 mm SL, with two rows of strong, densely arranged sharp-tipped conical tubercles anterior 5–6 branched rays of pectoral fin. Rostral barbel short, length of infraorbital 1 or to base of maxillary barbel; maxillary barbel much shorter, at most half length of rostral barbel.

Lateral line complete, along 30–33 body scales (Table 3) and 2 scales on caudal-fin base; comprising one tubed scale followed by a canal running steeply caudoventrad under unperforated scales to slightly posterior to pectoral-fin base, where curved caudad and represented by perforated scales running in a curve parallel to the ventral body outline and ending low on caudal peduncle and caudal-fin base; vertical section represented by about six scales; continued by two scales on caudal-fin base. Median predorsal scales 14 (2), 15 (10). Lateral scale rows passing between dorsal and pelvic fins ½6+1+2½ (7) ½7+1+2½ (5). Circumpeduncular scale rows 12 (12). A row of scales along anal-fin base. About ¼-1/3 of caudal-fin length scaled basally.

Dorsal- and anal-fin ray counts in Tables 4 and 5. Pectoral-fin rays i.10 (4), i.11 (6), i.12 (2). Pelvic-fin rays i.7 (12). Dorsal fin inserted at highest point of dorsum, little posterior to middle of body. Anal fin inserted below anterior rays of dorsal fin. Pectoral-fin insertion at about vertical through posterior margin of osseous opercle; pectoral fin not extending to pelvic-fin origin. Pectoral-fin axial lobe well developed. Pelvic fin inserted slightly anterior to mid-body, not reaching to anal-fin origin. Pelvic axillary scale present. Caudal fin forked, lobes of about equal length. Vertebrae 16+18 = 34 (2), 17+17 = 34 (1), 17+18 = 35 (6), 17+19 = 36 (1). Pharyngeal teeth 5,4,2/2,4,5 (NRM 65574, 66.2 mm SL).

#### Colouration in preservative

Dorsum pale grey, sides pale yellowish white, abdomen white. Opercle brownish or silvery depending on preservation. Sides of head sparsely pigmented. Narrow dark brown middorsal stripe from occiput to end of caudal peduncle. Dark brown or black, round or irregularly triangular cleithral spot covering part of first lateral-line scale and scale above. Along middle of side anterior to about vertical from anal-fin insertion a row of about 6–10 short dark vertical bars occupying a space equivalent to stripes P+1, P, and P-1. Anterior two bars greyish brown and narrow, followed by 4–5 wide, deep and contrasting black bars, preceding grey posterior bars. P stripe commencing posterior to bars, above anterior anal fin rays, extending to end of caudal peduncle; wide, black, with irregular borders, P stripe bordered dorsally by irregular narrow interstripe I. P+1 stripe dark brown, narrow, irregular, parallel with P stripe. P-1 stripe represented by indistinct spots detached from vertical bars may be present anteriorly on side, and present as 2–4 indistinct spots below P stripe. Dark markings predominantly brown in specimens preserved for a longer time, black in fresh specimens.

Dorsal fin pale grey basally, distal margin white. Anal fin pale grey basally, distal margin white. Pectoral and pelvic fins hyaline. Caudal fin semihyaline or pale grey; grey stripe along middle rays. Juveniles (Fig 3) with dark brown cleithral spot, about 5 pale vertical bars from about 20 mm SL; broad P stripe, indistinct P-1 stripe and contrasting interstripes I and I-1 posteriorly on side.

#### Geographical distribution

Known only from Bangladesh, recorded from coastal streams between Himchori and Teknaf, and the middle Sangu River upstream of Bandarban (Fig 3).

#### Comments

*Devario anomalus* was described from six large specimens, 57.0–81.0 mm SL, from south of Cox’s Bazar [9], probably at Himchori based on the coordinates in the original description. Data in the original description are confirmed by the present observations, except for the number of lateral line scales (33–35 according to [9]; 30–33 in our material), and different ranges of otherwise overlapping proportional measurements. *Devario anomalus* was already reported from Barachora and Kudung Cave [8]). The Donumia and Sangu localities are new records for the species, which is probably present in small streams in a wider area of the Cox’s Bazar district and Sangu River. Two specimens (CAS 94595) from the Sangu River that were reported [20] as not clearly identifiable as belonging to either *D*. *xyrops* or *D*. *anomalus*, fall within the variation of *D*. *anomalus* reported here. The sequenced Sangu specimens (NRM 67936–67937) do not depart morphologically from other *D*. *anomalus*, but the *COI* sequence corresponds to *D*. *aequipinnatus*.

*Devario anomalus* is most similar to *D*. *xyrops* from western Rakhine Yoma in Myanmar. They share a unique colour pattern in the genus with contrasting black bars in a group anteriorly, and a wide black P stripe posteriorly, separated by a few paler vertical bars. In *D*. *xyrops* the pale area is more obvious than in *D*. *anomalus*, however. Females of *D*. *anomalus* are more elongate than females of *D*. *xyrops* (body depth 26.3–30.2% SL vs. 31.4–35.8%; Fig 6). *Devario anomalus* also has a slightly narrower interorbital space (9.6–10.5% SL vs. 10.6–11.8%) than *D*. *xyrops*, a character which does not show sexual dimorphism. In the material available all male *D*. *xyrops* are larger than the male *D*. *anomalus*, and regression on standard length cannot be compared, but proportional measurements overlap (body depth 29.1–35.8% SL in *D*. *xyrops*, 25.9–30.2% in *D*. *anomalus*). Adult females of both species tend to be more deep-bodied than males but this is not expressed in proportional measurements, perhaps due to a sample bias because males are few and large males matching large females are available only for *D*. *xyrops*. The two largest specimens of *D*. *anomalus*, 96.7 and 79.2 mm SL (NRM 68131), are female and male, respectively, but not in good condition and not included in the measurements.

### Comparative morphometrics

**Fig 7:**
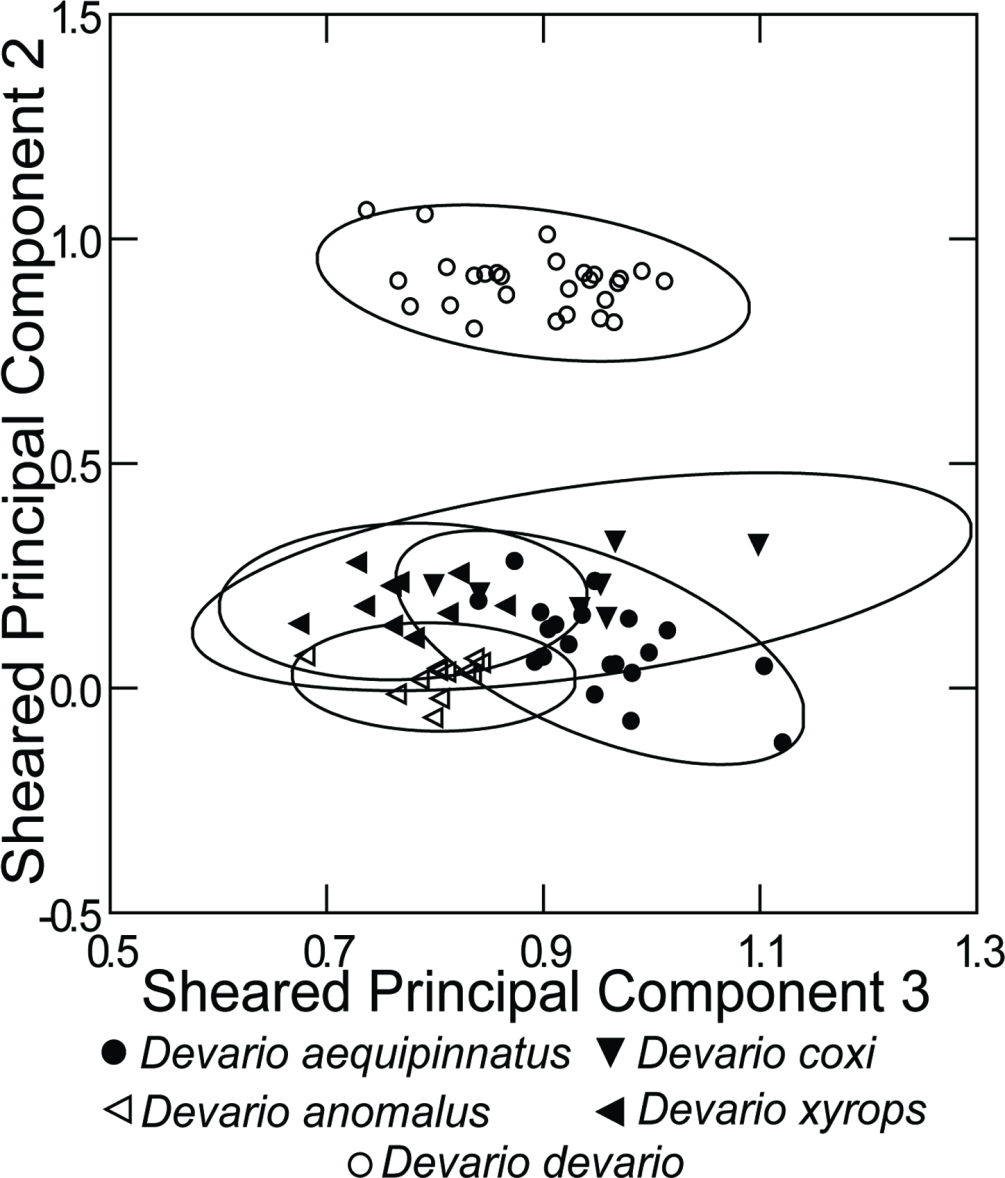
Plot of scores from principal component analysis of pooled measurements from *Devario aequipinnatus* from Bangladesh, *D*. *anomalus*, *D*. *coxi*, *D*. *devario* and *D*. *xyrops*.

**Fig 8:**
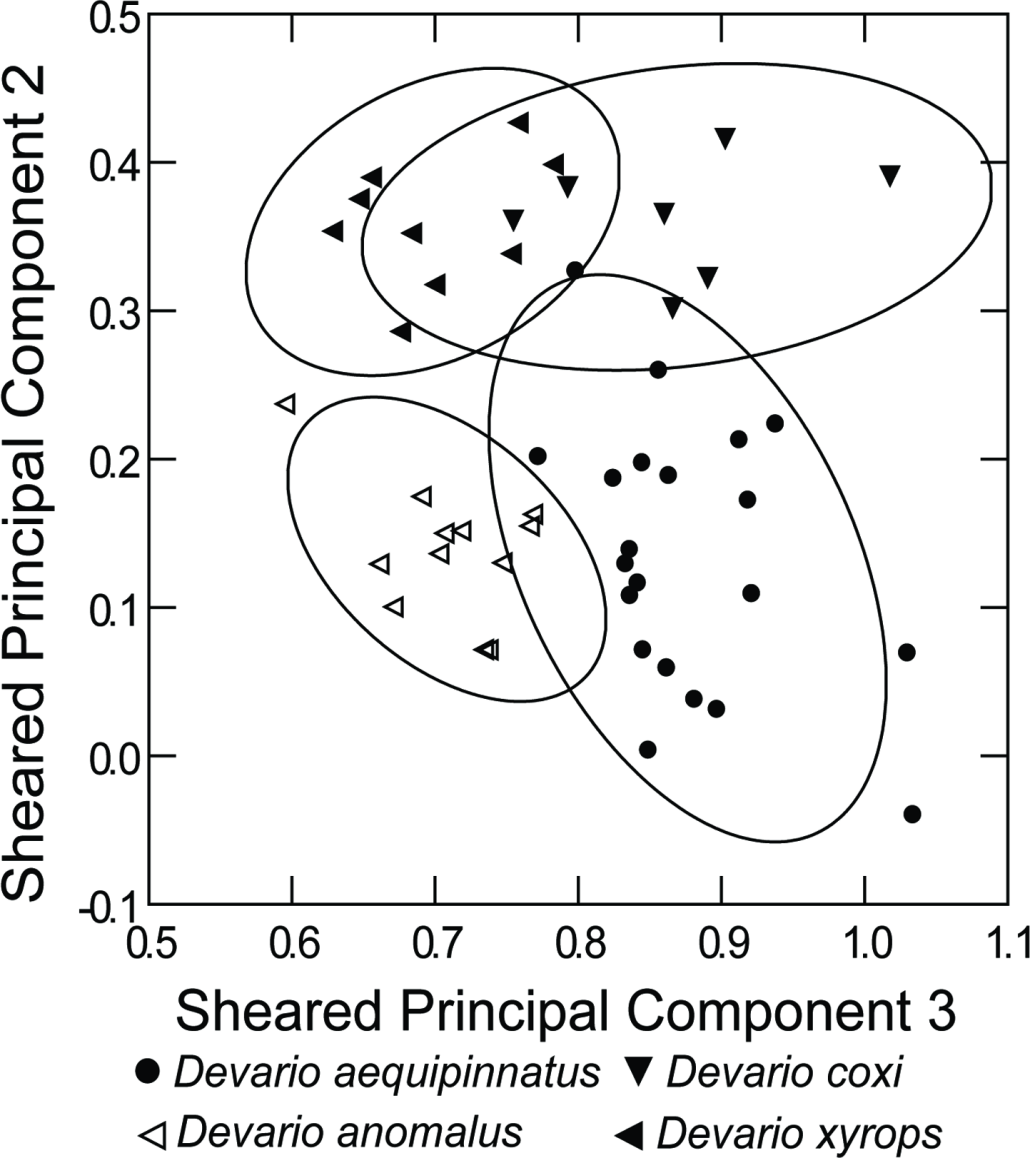
Plot of scores from principal component analysis of pooled measurements from *Devario aequipinnatus* from Bangladesh, *D*. *anomalus*, *D*. *coxi* and *D*. *xyrops*.

The principal component analysis of measurements from *D*. *aequipinnatus*, *D*. *coxi*, *D*. *anomalus*, *D*. *devario*, and *D*. *xyrops* (Table 9, Fig 7), separates *D*. *devario* from the rest based on a prominent PC2 in which the long dorsal-fin base is dominant. Removing *D*. *devario* results in more scatter (Table 10, Fig 8), probably marked by individual variability from different preservation and size ranges among species samples. Nonetheless, the PCA indicates different clusters for *D*. *anomalus* and *D*. *xyrops*, as well as for *D*. *aequipinnatus* and *D*. *coxi*.

**Table 9:** Component loadings in principal component analysis of pooled measurement data from *Devario aequipinnatus* from Bangladesh, *D*. *anomalus*, *D*. *coxi*, *D*. *devario* from Bangladesh and adjacent India, and *D*. *xyrops*.

Component	PC1	Sheared PC2	Sheared PC3	Sheared PC4
SL (mm)	0.238	-0.037	0.099	0.231
Body depth	0.249	0.344	0.302	-0.347
Head length	0.211	-0.056	-0.088	-0.086
Snout length	0.234	-0.210	-0.117	-0.105
Head depth	0.189	0.100	0.231	-0.027
Head width	0.236	-0.09	-0.061	-0.152
Upper jaw length	0.222	-0.38	-0.344	-0.114
Lower jaw length	0.199	-0.282	-0.308	-0.009
Orbital diameter	0.144	0.171	-0.001	-0.224
Interorbital width	0.225	-0.049	-0.103	-0.234
Caudal peduncle length	0.238	-0.420	0.379	0.449
Caudal peduncle depth	0.262	-0.043	0.111	0.013
Dorsal-fin base length	0.245	0.541	-0.190	0.320
Anal-fin base length	0.265	0.228	-0.465	0.453
Predorsal length	0.245	-0.161	0.108	0.014
Preanal length	0.234	0.047	0.275	0.064
Prepelvic length	0.227	0.057	0.234	0.023
Pectoral-fin length	0.226	0.011	-0.102	-0.344
Pelvic-fin length	0.239	0.078	-0.185	-0.182
Eigenvalue	0.610	N/A	N/A	N/A
Cumulative variance	77.3%	96.0%	96.9%	97.7%

**Table 10:** Component loadings in Principal Component Analysis of pooled measurement data from *D*. *aequipinnatus* from Bangladesh *D*. *anomalus*, *D*. *coxi*, *D*. *anomalus* and *D*. *xyrops*.

Component	PC1	Sheared PC2	Sheared PC3	Sheared PC4
SL (mm)	0.237	-0.127	0.114	-0.229
Body depth	0.263	0.382	0.263	0.153
Head length	0.209	-0.128	-0.085	0.255
Snout length	0.219	-0.213	-0.066	0.193
Head depth	0.194	0.056	0.295	0.151
Head width	0.232	-0.049	0.005	0.275
Upper jaw length	0.197	-0.356	-0.439	0.282
Lower jaw length	0.185	-0.102	-0.298	-0.045
Orbital diameter	0.153	0.297	0.132	0.321
Interorbital width	0.223	0.03	-0.051	0.326
Caudal peduncle length	0.220	-0.447	0.299	-0.256
Caudal peduncle depth	0.257	0.027	0.041	-0.169
Dorsal-fin base length	0.278	0.373	-0.162	-0.357
Anal-fin base length	0.287	0.084	-0.46	-0.33
Predorsal length	0.236	-0.135	0.051	-0.048
Preanal length	0.234	-0.055	0.258	-0.181
Prepelvic length	0.228	-0.017	0.233	-0.081
Pectoral-fin length	0.225	0.173	-0.067	0.236
Pelvic-fin length	0.243	0.373	-0.237	-0.065
Eigenvalue	0.845	N/A	N/A	N/A
Cumulative variance	95.3%	N/A	N/A	N/A

### Phylogenetic analysis and species delimitation

**Fig 9:**
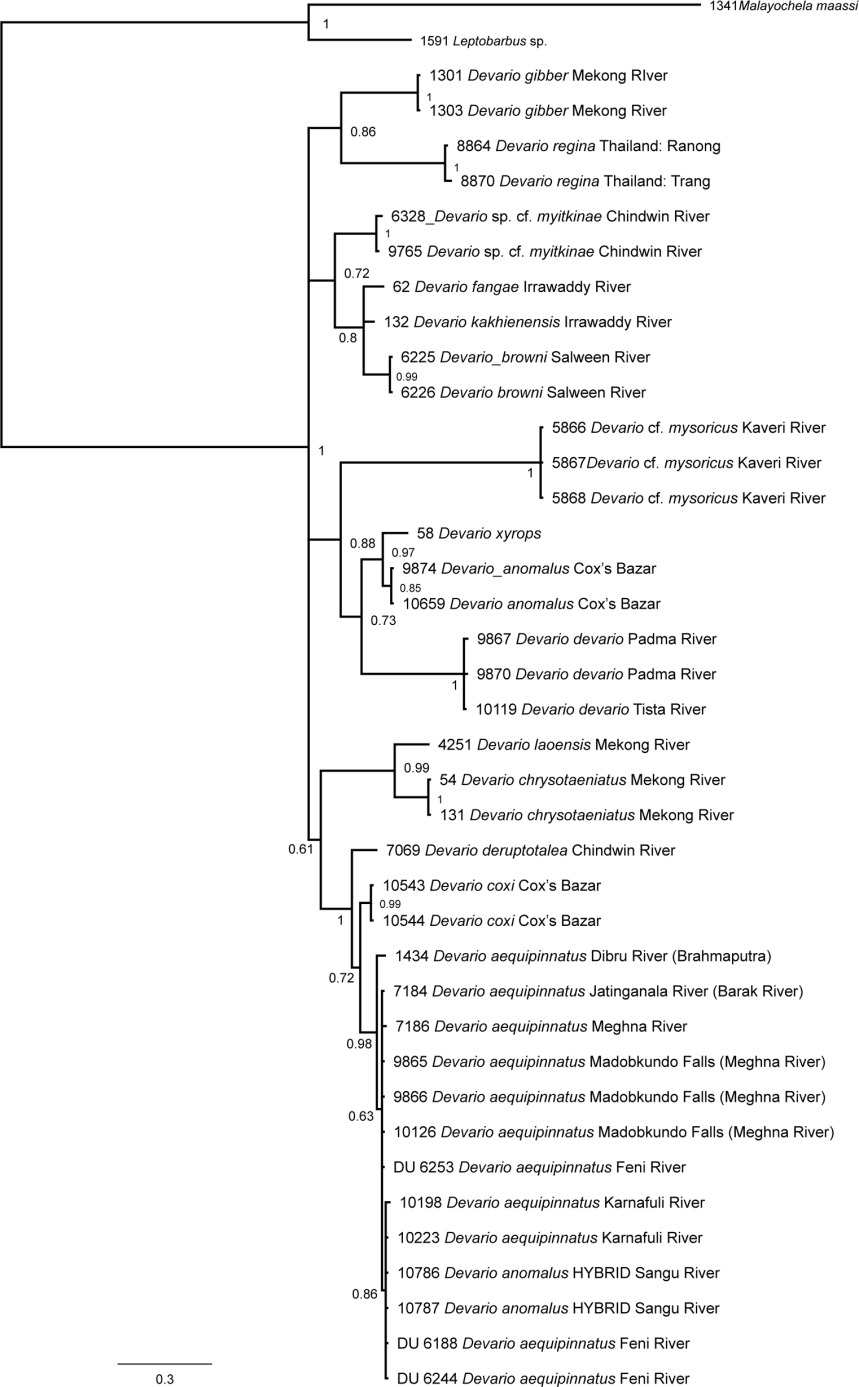
Phylogram of relationships of striped *Devario*, based on a Bayesian analysis of 655 bp fragment of the mitochondrial *COI* gene.

The phylogram based on *COI* sequences from a large number of striped species of *Devario* (Fig 9) is unresolved, with four distinct clades, (a) consisting of species from Thailand and Laos (Mekong River and Peninsular Thailand), (b) species from Myanmar (Irrawaddy and Salween Rivers), (c) a single species representing the Kaveri River, sister to a clade containing *D*. *anomalus*, *D*. *xyrops*, and *D*. *devario*); (d) striped species from the Brahmaputra, Chindwin, Feni, Karnafuli, Mekong, and Sangu Rivers, and the Majerchora stream near Cox’s Bazar, comprising *D*. *aequipinnatus*, *D*. *coxi* and *D*. *deruptotalea*.

**Fig 10:**
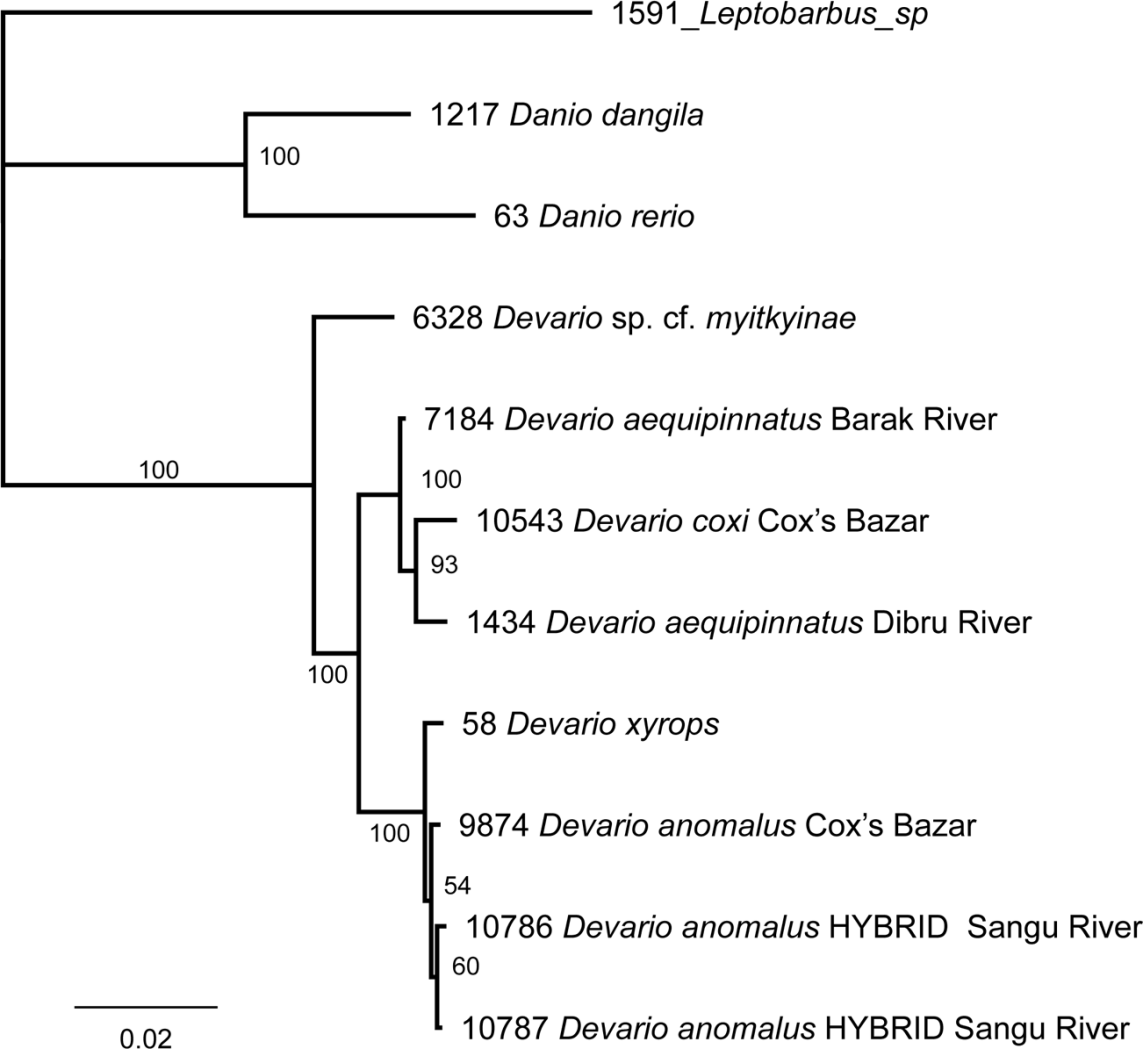
Phylogram of relationships of selected species of *Devario*, based on a Bayesian analysis of a 1565 bp fragment of the exon 3 of the nuclear *RAG 1* gene, targeting identity of specimens of *D*. *anomalus* with *D*. *aequipinnatus* mitogenome.

Two specimens of *Devario anomalus* from the Sangu River cluster with *D*. *aequipinnatus* in *COI* sequences (Fig 9, Table 11). In the nuclear *RAG 1* sequences (Table 1, Fig 10), those specimens are 99.5% similar to *D*. *anomalus* from near Cox’s Bazar, but only 97.3% similar to *D*. *aequipinnatus*.

**Table 11:** Closest uncorrected pairwise *p*-distances (percent dissimilarity) between species of striped *Devario* based on *COI* sequence data.

	1	2	3	4	5	6	7	8	9	10	11	12	13	14
(1) *D*. *aequipinnatus*														
(2) *D*. *coxi*	1.8													
(3) *D*. *deruptotalea*	2.6	2.0												
(4) *D*. *chrysotaeniatus*	7.2	7.6	7.2											
(5) *D*. *laoensis*	7.6	7.5	7.3	4.1										
(6) *D*. *browni*	6.7	7.6	6.7	8.2	8.9									
(7) *D*. *kakhienensis*	5.8	5.6	5.8	7.3	7.6	2.1								
(8) *D*. *fangae*	6.3	6.1	6.3	7.3	8.2	2.7	1.8							
(9) *D*. cf. *myitkyinae*	6.4	6.3	6.1	8.4	9	5.0	4.1	4.4						
(10) *D*. *anomalus*	6.4	6.6	6.7	8.9	7.6	7.2	6.6	6.7	7					
(11) *D*. *xyrops*	6.9	6.8	6.9	9.7	8.7	7.8	7.2	7.4	7.8	2.3				
(12) *D*. *devario*	8.1	8.2	8.1	11.0	10.7	9.8	8.4	8.5	8.7	7.3	7.7			
(13) *D*. *gibber*	8.6	9	9.2	10.1	9.4	7.8	7.8	8.1	8.9	9.2	9.8	11.5		
(14) *D*. *regina*	8.4	8.7	9.3	9.9	11.1	8.4	8.7	7.9	8.7	8.7	9.2	9.0	9.0	
(15) *D*. cf. *mysoricus*	11.6	10.5	11.5	14.4	13.6	11.5	11.8	11.6	12.4	10.8	11.8	12.5	12.6	12.8

Within species *p-*distances were either 0 (*D*. *anomalus*, *D*. *browni*, *D*. *chrysotaeniatus*) or 0.3% (*D*. *aequipinnatus*, *D*. *devario*, *D*. *gibber*), *D*. sp. cf. *myitkyinae* and *D*. sp. cf. *mysoricus)*, but with the exception of *D*. *aequipinnatus*, the samples are small.

### Other nominal species of *Devario* reported from Bangladesh

Ahmed et al. [8], reported *D*. *assamensis* from Bangladesh. Their photo, Fig 2, however, shows *D*. *aequipinnatus*. Ahmed et al. [8] reported *D*. *malabaricus* from Bangladesh. Their photo, Fig 2, however, shows *D*. *anomalus*. Conway et al. [9] discussed the status of *Perilampus ostreographus* M’Clelland, 1839 [13] in connection with Bangladeshi *Devario*, but did not have any specimens to demonstrate the existence of the species in Bangladesh. *Perilampus ostreographus* was synonymized with *Devario devario* by Day [39] and later authors.

### Key to *Devario* in Bangladesh

**1a** Infraorbital process and rostral barbel absent; 38–50 scales in lateral line; dorsal-fin rays iii.14½–17½…*D*. *devario*

**1b** Infraorbital process and rostral barbel present, 30–34 scales in lateral line, dorsal-fin rays iii.9½–12½; ……………………………………………..………………………………………….……2

**2a** Several short vertical bars in a group anteriorly on the side, the middle of the side with only indistinct vertical stripes, P stripe present posteriorly on the side to the base of the caudal fin…….*D*. *anomalus*.

**2b** P stripe uninterrupted from close to the head to the base of the caudal fin……. ……………… 3

**3a** Circumpeduncular scales 14, branched dorsal-fin rays 11½–12½, P and P-1 stipes confluent into dark field anteriorly, containing light spots …………………………….…………………….*D*. *coxi*

**3b** Circumpeduncular scales 12 (rarely 14), branched dorsal-fin rays 9½–10½ (rarely 11½), P and P-1 separate anteriorly ………………………………………………………………… *D*. *aequipinnatus*

## Discussion

### Taxonomy of *Devario aequipinnatus*

*Devario aequipinnatus* is one of the most commonly reported species of *Devario*, with numerous records from India, e.g., [17,39–40], some records from Nepal [9,37], and few, imprecise records from Bangladesh[11][35]. It is even reported from China, Myanmar, Pakistan, Sri Lanka, and Thailand [17]. Specimens reported from “rice paddy and creek east of Cox’s Bazar”[9] may represent *D*. *coxi*. *Devario aequipinnatus* was described by M’Clelland [13] as *Perilampus aequipinnatus* based apparently on a single preserved specimen that meanwhile probably has been lost. M’Clelland indicated that the specimen had been collected in Assam. At the time Assam covered a large part of northeastern India and northwestern Bangladesh. Since then, numerous species of *Devario* have been reported from South and Southeast Asia, many of them very similar to each other in appearance, and frequently synonymized with *D*. *aequipinnatus*, which name has served as a convenient container particularly for all species of *Devario* with a predominantly striped colour pattern.

M'Clelland’s 1839 monograph[13] on the Indian Cyprinidae was presented on 5 September 1838, and published in 1839. In the description of *Perilampus aequipinnatus* he makes special mention that his material was found among plants in spirits collected by William Griffith in Assam. He also mentions that “It has already been three years in spirits…” By twice referring to the studied material as “it” it seems likely that only a single specimen was available to M’Clelland. From the reference to Assam and that the specimen had been preserved since three years, it is obvious that it came from the expedition by M’Clelland, Wallich, and Griffith to Assam in 1835. This expedition left Pubna [Pabna, presently in Bangladesh], on 9 September 1835 and went, with many stops by way of Shiraz-gunge [Sirajganj], Jumalpore [Jamalpur], Mymensing [Mymensingh], the mouth of the Soormah [Surma River] and Hubbe-gunge [Habiganj], all presently in Bangladesh, north to Chattuc [Chhatak] and Khasiya mountains [Khasi Hills] in Churra Punjee [Cherrapunji], proceeding north along the Burrampooter [Brahmaputra] by way of Moflong [Mawphlang], Nunklow [Nonghlao], Guwahatti [Guwahati] and Tezpoor [Tezpur] to the region of Suddiya [Sadiya], from where the party returned in February 1836[41]. Around Sadiya, they stayed at Kujoo [Kujo Gaon], Negrigam [Ningru], Nadowar [Nadua], Rangangurrah [Rangagarrah], and Bangmara [Bengmara] and traveled on the Deboroo [Dibru River] and crossed the Maumoo stream [not located] [41]. The specimen of *D*. *aequipinnatus* could have been obtained in present-day Bangladesh as well as in any of the Indian localities visited by the expedition, although it seems more likely that it was obtained in the focus area of the expedition, in present-day Assam or Arunachal Pradesh.

The original description of *D*. *aequipinnatus* [13] is relatively brief, but compatible with an elongate species of *Devario*, except for the count of nine pelvic-fin rays. Remaining counts are recorded as D. 13, P. 13, A. 13, C. 19; lateral line scales 32; scale rows between dorsal and pelvic fins 8. There is no colour description, which is remarkable, as *D*. *aequipinnatus* has been identified by later authors as a species with distinctive striped colour pattern, and stripes are mentioned in the colour description and shown in the drawing of *D*. *ostreographus* in the same paper [13], p. 392, pl. 46 [= 45], Fig 3). The tongue is described as thick and corrugated, and whereas a tongue is absent in *Devario*, the skin covering the floor of the gape is thick with transverse grooves. M’Clelland also gives the length of the intestine and stomach, showing that the specimen was dissected. M’Clelland’s illustration [13], pl. 60, Fig 1, shows small dark spots in two or three rows on the side close to the back, but no other colour pattern.

There is no indication that the specimen described by M’Clelland is in existence. The identification of the fish as a species of *Devario* is not fully supported by an unambiguous autapomorphy, and the taxon should properly have been treated as a species inquirenda. Nevertheless, the widespread application of the name on a species of *Devario* merits its recognition.

The type locality area for *D*. *aequipinnatus*, as deduced from the itinerary of Griffith’s 1835 expedition includes present-day Bangladesh, and the Indian states of Meghalaya and Assam. We have not had access to specimens coming from specific places mentioned by Griffith[41] and listed above, but we base our concept of *D*. *aequipinnatus* on specimens from within the area covered by the general area of the expedition and which correspond to M’Clelland’s description except for probable differences in counting fin-rays, and the possession of a well-developed striped colour pattern. M’Clelland based *D*. *aequipinnatus* on the equal size of the dorsal and anal fins, probably referring to both having 13 fin rays. Although it is uncertain whether M’Clelland included or excluded the two shorter unbranched dorsal- and anal-fin rays in his count of 13, there are no alternative identifications than a species of *Devario*, as no other cyprinid genus in the area has partly opposed dorsal and anal fins with more than ten unbranched rays. In *Salmophasia*, *Barilius*, and *Opsarius* the dorsal fin, with usually seven branched rays, has a much shorter base than the anal fin, with more variation in number of fin rays, and the dorsal fin inserts well anterior to a vertical from the insertion of the anal fin; in *Chela*, *Laubuka*, and *Esomus*, with opposed dorsal and anal fins, the dorsal fin, with only 6–10 unbranched rays, has a much shorter base than the anal fin (data from [36]). In *D*. *aequipinnatus*, as diagnosed here, there are 1–2 more rays in the anal fin than in the dorsal fin, but there is no known species within the type locality area that comes closer to M’Clelland’s description.

Barman’s concept of *D*. *aequipinnatus* includes species that meanwhile have been considered as valid, i.e., *D*. *malabaricus* from southern India, *D*. *micronema* (Bleeker, 1863) from Sri Lanka, and *D*. *affinis* (Blyth, 1860), *D*. *browni* (Regan, 1907), and *D*. *strigillifer* (Myers, 1924) from Myanmar [1]. Talwar & Jhingran [36] synonymized *Danio deyi* Sen in Sen & Dey, 1985 [42], with *Devario aequipinnatus*. The deficient description and figures of *Danio deyi* suggest however, that it is a species of *Danio*, possibly *D*. *meghalayensis* Sen & Dey, 1985. The description of *D*. *meghalayensis*, however, has a photograph ([42], Fig II B) of a specimen looking like *Devario aequipinnatus* or *D*. *assamensis* but the quality does not permit a decision. The descriptions in [42] were discussed in [22].

Aside from *D*. *assamensis* and *D*. *ostreographus*, the only potential synonym of *D*. *aequipinnatus* is *Leuciscus lineolatus* Blyth, 1858, already synonymized by [39], followed by [17] and [1]. Blyth’s description [38], based on a single specimen from Darjeeling, agrees in scale and fin counts (D. 12, A. 14, P. 11, V. 8; 10 transverse scales, about 32 lateral line scales), and the colour markings mentioned correspond to the cleithral spot and P, P+1, and P-1 stripes, with interstripes: “A dusky spot behind the gill-covers, placed in a whitish space; beyond which a broad darkish band extends to the middle of the tail, bordered by a narrow pale line above and below, the lower not reaching so far forward as the upper: below this again another dark band and then white; and above a second and trace of a third pale line.” No type specimens are known to exist.

Jayaram [40] presented a review of *Devario malabaricus* and *D*. *aequipinnatus* from southern India, focusing on the diagnosis of *D*. *malabaricus*. The specimens he listed are from the Krishna, Kaveri (Coromandel Coast), and Bharathappuzha (Malabar Coast) drainages. His illustration of *D*. *aequipinnatus* shows a specimen from the upper Krishna River drainage, not far from Pune, agreeing with his view of *D*. *aequipinnatus* as a slender species with 31–34 lateral line scales. Two specimens, CMK 7250 with the same locality data represent a very slender species of *Devario*, with about 34 lateral-line scales, extremely short barbels, 12 circumpeduncular scales, infraorbital process absent; and a colour pattern including several short vertical bars anteriorly on the side, followed by horizontal stripes P, P+1 and P-1. It is consequently different from *D*. *aequipinnatus* in barbel development, absence of infraorbital process, and colour pattern. Several nominal species of *Devario* have been described from southern and western peninsular India, including *D*. *aurolineatus* (Day, 1865), *D*. *canarensis* (Jerdon, 1849), *D*. *fraseri* (Hora & Mukerji, 1935), *D*. *malabaricus*, *D*. *mysoricus* (Jerdon, 1849) and *D*. *neilgherriensis* (Day, 1867), all requiring taxonomic revision.

Numerous *COI* sequences identified as *Devario aequipinnatus* were downloaded from GenBank in August 2016. They reflect extensive barcoding projects currently underway in India. About half of those sequences do not concern the species here identified as *D*. *aequipinnatus*, but are from *D*. *malabaricus* and similar species in southern India. “Barcode sequences” from the Brahmaputra in Assam/Meghalaya (Genbank accession numbers FJ459485, KF511497–511499, KJ936744–48), the Tuicheng River, a tributary of the Kaladan River (Genbank accession numbers KJ936784–KJ936788) in Mizoram, and from Kamalganj, on the border with Tripura, apparently in the Meghna drainage (Genbank accession number KT364769) agree with our sequences of *D*. *aequipinnatus*. Based on preserved specimens and published barcode sequences, it seems that *D*. *aequipinnatus* is restricted to the Brahmaputra, Feni, Meghna, and Karnafuli basins, including the upper Tista River, and potentially present in the upper Kaladan drainage.

### Devario coxi

The striped *Devario* from Cox’s Bazar has a distinct *COI* haplotype different from all other species of *Devario* and is further distinguished from *D*. *aequipinnatus* and most other species of *Devario* by having 14 instead of 12 scales around the middle of the caudal peduncle, by addition of a scale row immediately below the dorsal median row. A few specimens of *D*. *aequipinnatus* from other localities in Bangladesh possess 14 circumpeduncular scales, but all *D*. *aequipinnatus* from India and Nepal have 12, the count present in almost all other species of *Devario*, and in the closely related genus *Betadevario*. Presence in the eastern Bangladeshi *D*. *aequipinnatus* of occasional specimens with 14 scales shows that the circumpeduncular scale count cannot be used alone as a diagnostic character of the *D*. *coxi*. The only other species of striped *Devario* with 14 circumpeduncular scales may be *D*. *acrostomus*. *Devario affinis*, *D*. *annandalei*, *D*. *spinosus*, *D*. *strigillifer*, and *D*. *yuensis*, which have very small scales, 35 or more in the lateral line, and 14–20 circumpeduncular scales. *Devario coxi* is phenotypically distinct also in more branched dorsal-fin rays, but with narrow overlap (Table 4). The colour pattern is slightly different as in *D*. *aequipinnatus* the P, P+1 and P-1 stripes are integer, with the exception that the I-1 stripe may be interrupted anteriorly (Fig 4A–4C); whereas in *D*. *coxi* the P+1 stripe is irregular anteriorly, and the P and P-1 stripes are joined anteriorly on the side in a wide dark area incorporating light spots (Fig 4D and 4E). *Devario coxi* is represented by large adults, and *D*. *aequipinnatus* by many small specimens, rendering a morphometric comparison difficult, as size allometry may influence proportional measurements. Nevertheless, the PCA (Fig 8) supports distinct morphologies. Although very similar to *D*. *aequipinnatus*, genetic distinctness, fin counts, scale counts, colouration and morphometrics combined provide evidence for recognition of *D*. *coxi* as a distinct species.

Pairwise *p*-distance is the proportion of differences, or divergence, between two DNA sequences, expressed in percent. As a coarse empirical rule-of-thumb, for the mitochondrial *COI* gene, the pairwise p-distance between any two members of the same species is typically <1%, while the pairwise p-distance between members of different species is typically ≥2% [43], and can be used as an indicator of species affinity.

The *p-*distance among striped *Devario* range from 1.8 to 12.8 (Table 11). The distance between *D*. *aequipinnatus* and *D*. *coxi* is 1.8%, which is low for the genus but similar to other closely related similar species like *D*. *kakhienensis* and *D*. *fangae* which occur in separate headwaters of the Irrawaddy River in Myanmar and China. At the other end, *D*. *deruptotalea* is more similar (98%) to *D*. *coxi*, than to *D*. *aequipinnatus* (94%), conforming to the close relationship in the phylogenetic analysis. Rather than insisting on a rule of thumb of 2% *p-*distance as species criterion, we accept the morphologically differentiated *D*. *aequipinnatus*+*D*. *deruptotalea*+*D*.*coxi* and *D*. *browni*+*D*. *fangae*+*D*.*kakhienensis* as two groups of closely related species.

Masters et al. [29] proposed P ID(Liberal), a statistical comparison of the distribution of intraspecific variation within a putative species to the interspecific distance and intraspecific variation within its closest relative, which estimates the mean (95% confidence interval) probability of correctly identifying an unknown member of the putative species, with the criterion that it should fall within or sister to the putative species. P ID(Liberal) supports the distinctiveness of the *D*. *coxi* (0.97).

Monophyletic groups in gene trees can be artefacts caused by stochastic gene coalescence within a larger panmictic group. Rosenberg’s P(AB) statistic [44] is an estimate of the probability that a putative species is such a randomly monophyletic clade relative to its closest sister clade under a null model of random coalescence. Rosenberg’s P(AB) supports *D*. *coxi* as a monophyletic group separate from *D*. *aequipinnatus*.

The *RAG1* sequence of *D*. *coxi* is identical to that of *D*. *aequipinnatus* (Fig 10), and seems likely that the two represent relatively recently isolated species.

### Other species of *Devario* reported from Bangladesh

*Devario assamensis* was described by Barman [18] on the basis of three specimens from Tangla, Darrang district, Assam, characterized by a very deep body, (2.87–3.1 times in SL), 40–41 lateral-line scales, dorsal fin rays ii.12, anal-fin rays ii.16–17. It was synonymized with *D*. *regina* by Talwar & Jhingran [36] without explanation. Tilak & Jain [19] attempted to correct Barman’s description of the type specimens of *D*. *assamensis*. The only significant difference, however, seems to be their count of 36 instead of 40–41 scales in the lateral line, and presence instead of absence of a supraorbital process (“pointing forward above the anterior superior margin of the orbit”). Unpublished notes on the holotype made by the late Fang Fang mention an infraorbital process, but no supraorbital process and it is not showing on her photograph of the holotype. It may be that the supraorbital process observed by Tilak & Jain is the anterior projection of the orbital shelf formed by the supraorbital, or a sharp curved process of the lateral ethmoid supporting the nasal cavity. The latter is frequently exposed in poorly preserved or desiccated specimens of *Devario*. The supraorbital process in *Devario spinosus*, referred to by Tilak & Jain, is a flat, blunt, erect projection from the supraorbital (pers. obs.). We count 34/35±1 scales in the lateral line and 34 scales along the middle of the side on the photograph of the holotype of *D*. *assamensis*, and at least 12½ or 13½ branched anal-fin rays. The holotype has lost most of the caudal fin and also the other fins are damaged. It shows only faint traces of the colour pattern, but it is consistent with the description and photograph by Barman [18], pl. 84, including P+1, P, and P-1 stripes. The colour pattern is thus similar to that of *D*. *aequipinnatus*. With the reservation that both Barman’s and Tilak & Jain’s counts may be incorrect (e.g., 2 instead 3 of unbranched rays in the dorsal and anal fins), their count of 16–17 (vs. 11.5–13½ branched anal-fin rays, and 36 (vs. 30–33) lateral line scales suggest that *D*. *assamensis* may be a distinct species. *Devario assamensis* is so far known only from the type series.

Conway et al. [9] discussed the status of *Perilampus ostreographus* M’Clelland [13] in connection with Bangladeshi *Devario*, removing it from the synonymy of *D*. *devario*. M’Clelland gave *P*. *ostreographus* a wide distribution which potentially includes present-day Bangladesh: “throughout Assam in small indolent streams, as well as in the larger rivers, … also abundant in some parts of Bengal.” The species was referred to *Devario* by Heckel [45], p. 1015, as *D*. *osteographus*. *Devario cyanotaenia* Bleeker, 1860, is a junior objective synonym, based on M’Clelland’s [13] drawing of *D*. *ostreographus*. No type specimens are known to exist. According to the data in the original description and the accompanying figure, reproduced by Conway et al. [9], Fig 6, it is a deep-bodied, striped danionin, characterized by M’Clelland by body depth almost equal to half of the total length (to be compared with body depth one-third to one-fourth of the total length in his description of *D*. *aequipinnatus*), mouth and head directed upwards; 35 scales along the sides, 15 scales in a transverse row; fin counts D. 12, A. 16, P. 15, V. 8, C. 19; colour pattern with purple stripes along the side “to the extremity of the caudal”; prominent knob at the tip of the lower jaw; tongue thick and corrugated; and “caudal almost entire, the middle rays being very little shorter than the outer”. The pectoral and pelvic fin counts are probably incorrect, as there are rarely more than 12 branched pectoral-fin rays in *Devario*. The description of the colour pattern is compatible with striped species of *Devario*, except that in *Devario* the dark stripes along the side do not continue on the caudal fin, except for the continuation of the P stripe. The image, however does not show any stripes on the caudal fin. In *Danio* the stripes on the side of the body may be continued on the caudal fin, as in *D*. *dangila* and *D*. *rerio*. With a size of “usually about three inches” (76 mm), *P*. *ostreographus* is too large for *D*. *rerio*. Adult *Devario* always possess a distinctly emarginate caudal fin, whereas the caudal fin becomes almost subtruncate in large specimens of *Danio*. The trunk colour pattern of *D*. *dangila* and other members of the chain danio group is different from that of *D*. *ostreographus* in being composed of a combination of narrow stripes and contiguous rings. M’Clelland does not mention presence of barbels, suggesting that the species does not have barbels, or only very short barbels, whereas most species of *Danio* have very long barbels. It is clearly different from the other deep-bodied *Devario* in Bangladesh and northern India, *D*. *devario*, in presence of multiple flank stripes and shorter dorsal fin (12 vs. 14½–17½ branched rays). Based on the colour pattern and the fin counts, and the option that *P*. *ostreographus* represents large deep-bodied females, it is potentially the same species as *D*. *aequipinnatus*. Both species were described in the same paper, but have never before been considered to be the same species. As First Revisers we give precedence of priority to *Perilampus aequipinnatus*, under the International Code of Zoological Nomenclature, Article 24.2.[46]. We do not think it is necessary to fix a neotype for *P*. *ostreographus*, as the synonymy is tentative and the fish fauna of its area of occurrence is still incompletely known. There is at least no indication of occurrence in Bangladesh of a deep-bodied species of *Devario* conforming to the description by M’Clelland [13].

Because *D*. *ostreographus* and *D*. *assamensis* are the only known deep-bodied *Devario* known from the Ganga-Brahmaputra basin besides *D*. *devario* it seems possible, potentially supported by the similarity in reported shape and colour pattern, and anal-fin count, that *D*. *assamensis* is a junior synonym of *D*. *ostreographus*, and in extension, a synonym of *D*. *aequipinnatus*. The 1835 expedition to Assam, in which McClelland participated [41], passed Darrang on the way, and it is possible that *P*. *ostreographus* was collected in that part of the Brahmaputra.

No specimens identified by McClelland as *P*. *ostreographus* are known to have been preserved. Four specimens of *D*. *devario* in SMF 8835, donated by McClelland in 1844, are in a very bad state of preservation but are definitely referable to *D*. *devario*. Associated collection information does not indicate that they represent *D*. *ostreographus* (SMF, pers. comm.) The species is not listed in the catalogue of type specimens in the Zoological Survey of India, Calcutta [47].

Day [48] synonymized species currently in *Devario* (*Danio micronema*, *Devario cyanotaenia*, *Perilampus canarensis*, *P*. *malabaricus*, *P*. *mysoricus*) under the name of *Danio osteographus* without explanation, and later [39], he synonymized *D*. *ostreographus* with *Devario devario*, and synonymized the other species under *D*. *malabaricus*. Later ichthyologists followed Day's authority, and all treated *D*. *ostreographus* as a junior synonym of *D*. *devario* [1], [17], [36], [49], until Conway et al. [9] re-validated *D*. *ostreographus* as a species without known specimens.

Barman [17] considered *D*. *malabaricus* to be a synonym of *D*. *aequipinnatus*, but Talwar & Jhingran [35] used the name as valid for a species with rudimentary barbels. Jayaram [40] attempted to distinguish *D*. *malabaricus* by slightly higher scale count (35–38; material from Andhra Pradesh, Karnataka, Maharashtra and Kerala) than in *D*. *aequipinnatus* (31–34; material from Maharashtra and Tamil Nadu), but it in the absence of types the identification remains spurious. Jayaram considered Madras (now Chennai) as type locality of *D*. *malabaricus*. However, in the introduction to the paper containing the original description, Jerdon [50], p. 139, notes that his "…own researches have been, as yet, confined to parts of the Carnatic, of Mysore, and of Malabar.” Jerdon [51], p. 325 gives the locality of *P*. *malabaricus* as “…common in all the streams of Malabar out of reach of the tides…”. Malabar should be understood as the Malabar Coast, i.e., a coastal region of southern India west of the Western Ghats, or the Malabar district, within that area (currently in the Indian state of Kerala). Jerdon [51], p. 325, described two other species of *Devario* with geographical names, viz., *P*. *canarensis* from streams of Canara (Indian coast north of the Malabar Coast), and *P*. *mysoricus* from the Kaveri River (east of the Western Ghats, within the historical Mysore region). These nominal species have been synonymized with *D*. *malabaricus* [1], but have not been subject to a critical taxonomic assessment.[3] proposed a common neotype for *D*. *malabaricus* and *D*. *mysoricus*, but failed to demonstrate an exceptional need for a neotype as required by [46], Articles 75.2–3. There is no indication that *D*. *malabaricus* or any other southern Indian species of *Devario* would be present in Bangladesh.

### Phylogenetic relationships

The phylogram, Fig 9, includes sequences from vouchered specimens of all species of striped *Devario* available to us. The ingroup is contained in a polytomy with four unresolved clades with some geographical correlation. *Devario aequipinnatus* from different localities show only very slight variation; *D*. *coxi* is sister to *D*. *aequipinnatus*, and the sister species to *D*. *aequipinnatus+D*. *coxi* is *D*. *deruptotalea* from the Chindwin River in Manipur. The sister group of *D*. *aequipinnatus+D*. *coxi+D*. *deruptotalea* consists of two species from the Mekong River, which is unexpected, but the node support is low.

*Devario xyrops* and *D*. *anomalus* are resolved as sister taxa, as expected from their morphological similarity, but their sister group relation to *D*. *devario* is unexpected. *Devario* cf. *mysoricus* represents a southern peninsular group also including *D*. *malabaricus*, for which there was no relationship hypothesis formulated earlier, but which was usually synonymized with *D*. *aequipinnatus*. A similar but slightly different subtree was found by Tang et al. [52], Fig 1, with *D*. *anomalus* and *D*. *xyrops* as sister taxa, and sister to a clade consisting of *D*. *devario*, *D*. *pathirana* and *D*. cf. *malabaricus*.

Two clades combine Mekong+Peninsular Thailand (*D*. *gibber* and *D*. *regina*), and Myanmar species (*D*. *fangae*, *D*. cf. *myitkyinae*, *D*. *kakhienensis*, *D*. *browni*). The latter group consists of relatively similar species distributed in northern Myanmar, with slight morphological differences between different river basins.

Two individuals from the Sangu River morphologically identified as *Devario anomalus* were found to have identical *COI* sequences to *Devario aequipinnatus*.

*Devario anomalus* is resolved as sister species the very similar *D*. *xyrops*, separated by 2.3% p-distance, supporting the view that *D*. *xyrops* is distinct from *D*. *anomalus*.

### Introgression

In the *COI* phylogram, Fig 9, two specimens morphologically identified as *D*. *anomalus* (tissue vouchers 10786 and 10787) are found in the *D*. *aequipinnatus* clade, distant from the remaining *D*. *anomalus*. These two specimens differ from *D*. *aequipinnatus* by 0.3% or less, strongly indicating that they are conspecific. However, their nuclear *RAG1* sequences place them with other *D*. *anomalus* (Fig 10), 0.5% dissimilar from the other *D*. *anomalus*, and 2.7% dissimilar from *D*. *aequipinnatus*. These two specimens consequently represent introgression of *D*. *aequipinnatus*. This seems to be the first case of hybridization in the wild between two species of danionine cyprinids. Hybridization is reported from many other cyprinids, but then mainly from Europe and the United States and usually in anthropogenic conditions [53], and no documented case of hybridization in nature in Asia. The present case is noteworthy because there is no record of *D*. *aequipinnatus* from the Sangu River. The closest known populations of *D*. *aequipinnatus* are in the Karnafuli drainage. It is not impossible that *D*. *aequipinnatus* has been overlooked in the Sangu drainage, or the introgression is due to an ancient river connection and faunal exchange.

### Biogeography

Bangladeshi species of *Devario* show a distinct pattern by presence and absence data. Most noteworthy is that *Devario devario* is absent from southeastern Bangladesh, although there are suitable habitats in the major rivers, the Karnafuli and Sangu. There are also no collecting records from the Sundarbans or elsewhere in the lowland southwestern portion of Bangladesh. Outside Bangladesh the geographical range of *D*. *devario* stretches along the Brahmaputra up to Dibrugarh, and in the Ganga River basin in Nepal and India west to the Indus River drainage [17][54].

*Devario aequipinnatus* turns out to be a widespread species in the Brahmaputra drainage in northeastern India, eastern Nepal, and in Bangladeshi rivers Meghna, Feni and Karnafuli; but absent from the Sangu River and coastal streams, except for the presence of *D*. *aequipinnatus* mtDNA in *D*. *anomalus* in the Sangu River. This is still a much more restricted distribution than claimed by, e.g., Barman [17].

The sister-group relationship of *D*. *anomalus* and *D*. *xyrops*, the latter endemic to the western slope of the Rakhine Yoma—and the only species of *Devario* present there—suggests allopatric speciation, although there are no obvious geographical barriers between the Cox’s Bazar and Teknaf region and the Rakhine Yoma, except possibly the large Kaladan River, which forms the border between India and Myanmar, emptying in the Bay of Bengal at Sittwe. The collecting record of *D*. *xyrops* and *D*. *anomalus* is still meagre, and should be expanded in order to provide material for a biogeographical analysis.

*Devario aequipinnatus* is a widespread species in northeastern India and Bangladesh, including the Feni and Karnafuli Rivers, but absent from the Sangu River and coastal streams, except for the distinctive population near Cox’s Bazar, and the presence of *D*. *aequipinnatus* mtDNA in *D*. *anomalus* in the Sangu River.

Whereas the collecting records of *Devario* definitely indicate southeastern Bangladesh—with two endemic species—as distinct from the northern and western parts of the country, and possibly pertaining to the distinctive fauna of the Rakhine Yoma, the geographical coverage of available collections is still limited. The presence of *D*. *devario* and *D*. *aequipinnatus* in the rest of the country represents a portion of two widely distributed species in the Brahmaputra basin. Published inventories (e.g., [8]) nevertheless suggest that the four species of *Devario* reported here represents the total presence of the genus in Bangladesh.
